# Missense mutations in CRX homeodomain cause dominant retinopathies through two distinct mechanisms

**DOI:** 10.7554/eLife.87147

**Published:** 2023-11-14

**Authors:** Yiqiao Zheng, Chi Sun, Xiaodong Zhang, Philip A Ruzycki, Shiming Chen

**Affiliations:** 1 Molecular Genetic and Genomics Graduate Program, Division of Biological and Biomedical Sciences, Washington University in St Louis Saint Louis United States; 2 Department of Ophthalmology and Visual Sciences, Washington University in St Louis Saint Louis United States; 3 Department of Genetics, Washington University in St Louis Saint Louis United States; 4 Department of Developmental Biology, Washington University in St Louis Saint Louis United States; University of Chicago United States; New York University United States

**Keywords:** homeodomain, transcription factor, DNA-binding specificity, CRX disease mutations, inherited retinal diseases, photoreceptor development, Mouse

## Abstract

Homeodomain transcription factors (HD TFs) are instrumental to vertebrate development. Mutations in HD TFs have been linked to human diseases, but their pathogenic mechanisms remain elusive. Here, we use *Cone-Rod Homeobox* (*CRX*) as a model to decipher the disease-causing mechanisms of two HD mutations, p.E80A and p.K88N, that produce severe dominant retinopathies. Through integrated analysis of molecular and functional evidence in vitro and in knock-in mouse models, we uncover two novel gain-of-function mechanisms: p.E80A increases CRX-mediated transactivation of canonical CRX target genes in developing photoreceptors; p.K88N alters CRX DNA-binding specificity resulting in binding at ectopic sites and severe perturbation of CRX target gene expression. Both mechanisms produce novel retinal morphological defects and hinder photoreceptor maturation distinct from loss-of-function models. This study reveals the distinct roles of E80 and K88 residues in CRX HD regulatory functions and emphasizes the importance of transcriptional precision in normal development.

## Introduction

Homeodomain transcription factors (HD TFs) play a fundamental role in vertebrate development. Members of the HD TF family are characterized by the presence of a highly conserved 60 amino acid helix-turn-helix DNA-binding domain known as the homeodomain (HD). The HD is one of the most studied eukaryotic DNA-binding motifs since its discovery in *Drosophila* homeotic transformations (Mark et al., 1997; Lewis, 1978). Hundreds of HD TFs have subsequently been documented in regulating gene expression programs important for body plan specification, pattern formation, and cell fate determination (Mark et al., 1997; Lewis, 1978). Mutations in HD TFs have been linked to many human diseases, including neuropsychiatric and neurodegenerative conditions (Leung et al., 2022; Chi, 2005). Although significant progress has been made in understanding HD–DNA interactions, uncovering the pathogenetic mechanisms of disease-causing missense mutations in HD have proven challenging.

The retina has long been used as a model system to study the role of HD TFs in normal central nervous system development and in neurological diseases (Zagozewski et al., 2014). During retinogenesis, HD TFs play essential roles in the patterning of neuroepithelium, specification of retinal progenitors and differentiation of all retinal cell classes that derive from a common progenitor (Diacou et al., 2022). Importantly, many HD TFs are shared between the brain and the retina during development and mutations in these TFs can lead to disease manifestation in both tissues (Beby and Lamonerie, 2013; Henderson et al., 2009; Abouzeid et al., 2009; Lima Cunha et al., 2019; Voronina et al., 2004; Abouzeid et al., 2012). The accessibility and wealth of available molecular tools make the retina a valuable tool to decipher the pathogenic mechanisms of HD TF mutations associated with neurological diseases.

Here, we study CRX, a HD TF essential for photoreceptor cells in the retina, as a model to understand how single amino acid substitutions in the HD impact TF functions and cause blinding diseases. Photoreceptors are the most numerous neurons in the retina and are specialized to sense light and initiate vision through a process called phototransduction. Animal studies have demonstrated that *Crx* is first expressed in post-mitotic photoreceptor precursors (Muranishi et al., 2011) and maintained throughout life (Chen et al., 1997; Furukawa et al., 1997). Loss of CRX results in impaired photoreceptor gene expression, failure of maturation and rapid degeneration of immature, non-functional photoreceptors (Furukawa et al., 1999). Protein-coding sequence variants in human *CRX* have been associated with inherited retinal diseases (IRDs) that affect photoreceptors: Leber congenital amaurosis (LCA), cone–rod dystrophy (CoRD), and retinitis pigmentosa (RP) (OMIM:602225). However, the recessive phenotype observed in *Crx* knockout mouse models fails to recapitulate many dominant human *CRX* mutations that arise de novo *(*Furukawa et al., 1999).

**Figure 1.**
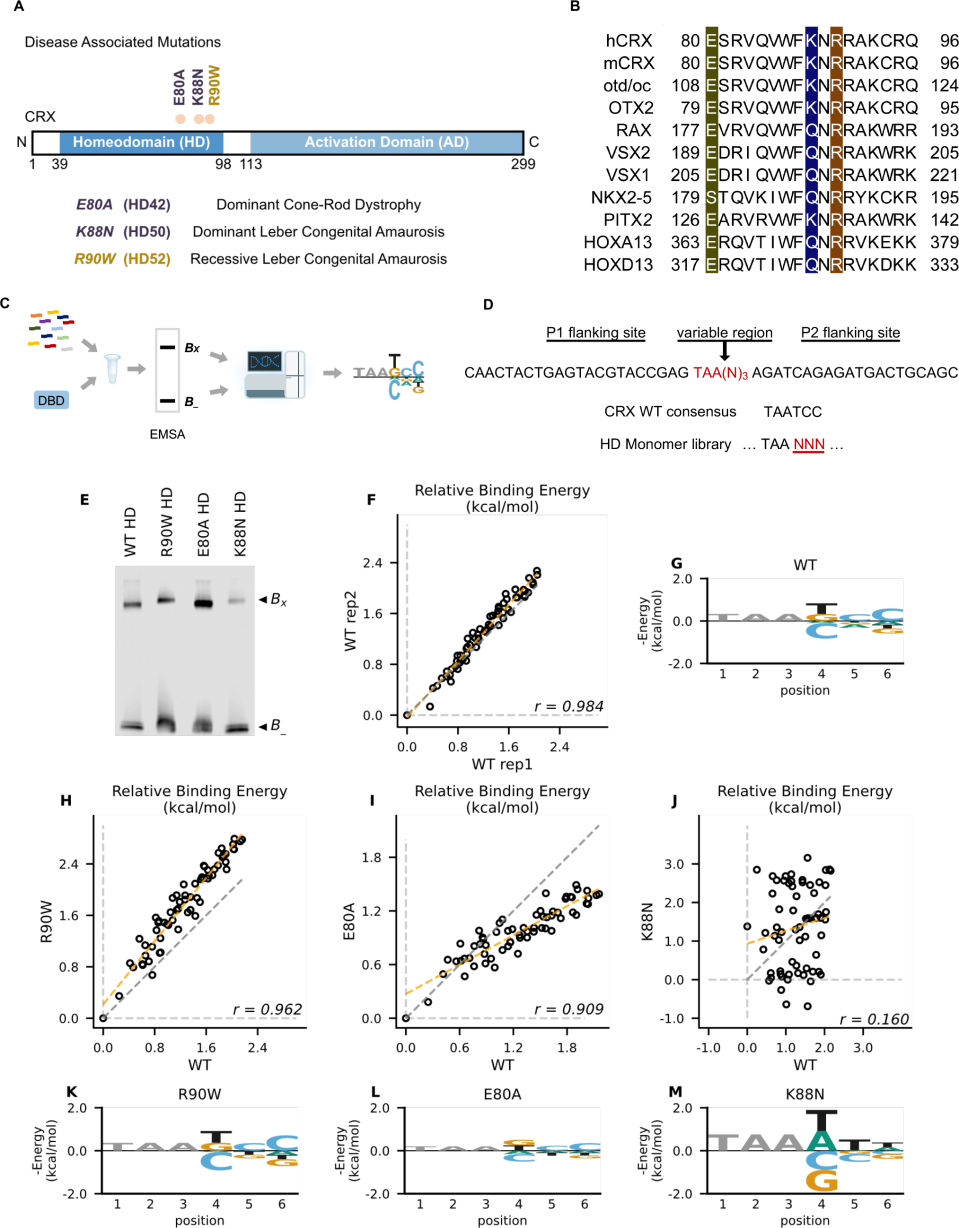
Disease associated missense mutations altered CRX homeodomain (HD) DNA-binding specificity (legend on the next page). (**A**) Diagram of CRX functional domains: HD for DNA-binding and activation domain (AD) for target gene transactivation. The three missense mutations in this study are located at the C-terminus of CRX HD and associated with different retinal diseases in human. Number in the parenthesis denotes the CRX HD position of the corresponding mutated residue. (**B**) Alignments of HD recognition helix sequences for the indicated HD proteins for which HD missense mutations have been associated with inherited diseases. Accession numbers can be found in Supplementary file 1a. Missense variants in this study (highlighted) are located at highly conserved residues across species and different HD transcription factors (TFs). (**C**) Spec-seq experimental workflow (Methods). (**D**) Spec-seq library design of monomeric HD-binding sites. (**E**) Electrophoretic mobility shift assay (EMSA) gel images of Spec-seq experiments with different CRX HD species. B_x_: bound. B_-_: unbound. (**F**) Relative binding energy comparison from two different experiments with wild-type (WT) HD. (**G**) Binding energy model for WT CRX HD. Relative binding energy comparison between WT HD and R90W HD (**H**), E80A HD (**I**), or K88N HD (**J**). Consensus sequence is defined to have relative binding energy of 0kT (TAATCC for WT, R90W and E80A, TAATTA for K88N). The identity line is represented in gray dash. The orange dashed line shows the best linear fit to the data. Binding energy models for R90W HD (**K**), E80A HD (**L**), and K88N HD (**M**). Only sequence variants within two mismatches to the corresponding consensus sequences were used to generate binding models. Negative binding energy is plotted such that bases above the *x*-axis are preferred bases and bases below the *x*-axis are unfavorable bases. Constant bases (TAA) carried no information are drawn at arbitrary height in gray. Figure 1—source data 1. Figure 1—source data 2.

CRX contains two functional domains – the N-terminal HD and C-terminal activation domain (AD) (Figure 1A), both are required for proper activation of target genes and maintenance of normal *Crx* mRNA transcript abundance (Chau et al., 2000; Chen et al., 2002). To understand how CRX HD mutations cause diseases, we have previously reported a mutation knock-in mouse model carrying a hypomorphic mutation p.R90W (R90W) in CRX HD (Tran et al., 2014; Ruzycki et al., 2015). We found that R90W mutation produces a recessive loss-of-function phenotype very similar to that of *Crx* knockout mice.

**Figure 1—figure supplement 1.**
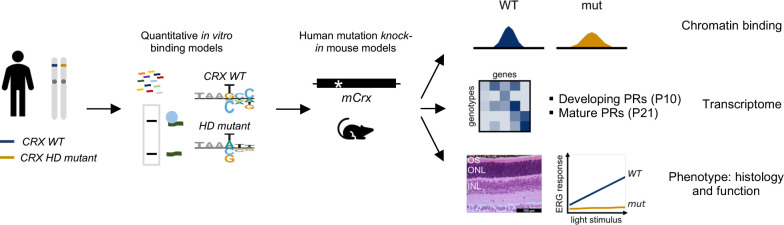
Multi-omics approach to investigate the functional consequences of dominant disease mutations on CRX regulatory activities and photoreceptor development. Human retinopathy associated CRX homeodomain (HD) mutant is first tested in vitro for HD–DNA interactions by Spec-seq. Quantitative binding models are generated for wild-type (WT) and mutant HDs. Each mutation is then introduced into endogenous *mCrx* locus to generate human mutation knock-in mouse models. ChIP-seq is employed to characterize CRX chromatin binding in WT and mutant mouse retinas. Bulk RNA-seq is then applied to determine transcriptomic changes in developing (P10) and mature (P21) photoreceptors (PRs) in both WT and mutant mouse retinas. Last, phenotypic characterization on retinal morphology and visual functions is carried out to understand the consequences of mutant CRX chromatin binding and associated transcriptomic alterations.

**Figure 1—figure supplement 2.**
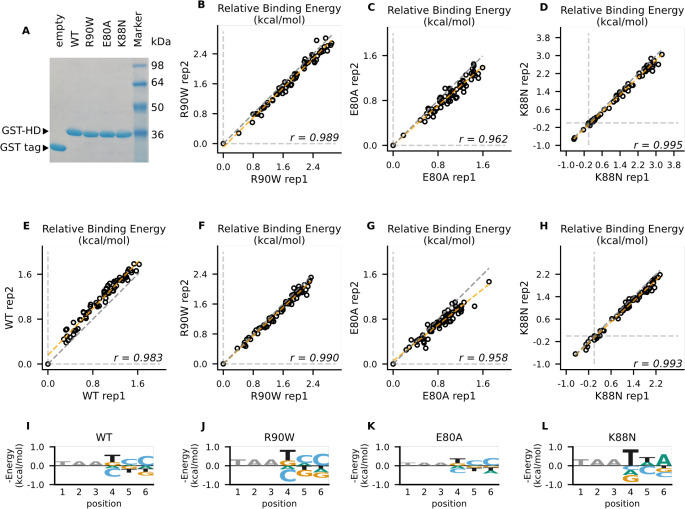
Reversed-strand Spec-seq library showed similar changes in mutant CRX homeodomain (HD) DNA-binding specificity. (**A**) Native sodium dodecyl sulfate–polyacrylamide gel electrophoresis (SDS–PAGE) gel image of affinity-purified empty GST tag and GST-CRX HDs. Relative binding energy comparison from two different experiments for R90W HD (**B**), E80A HD (**C**), and K88N HD (**D**) on the same Spec-seq library as in Figure 1. (**E–L**) Spec-seq experiments of a second library with the TAANNN sites on the reverse strand show similar results. Relative binding energy comparison from two different experiments for wild-type (WT) HD (**E**), R90W HD (**F**), E80A HD (**G**), and K88N HD (**H**) on the reversed monomeric library. The identity line is represented in gray dash. The orange dashed line shows the best linear fit to the data. Binding energy models for WT HD (**I**), R90W HD (**J**), E80A HD (**K**), and K88N HD (**L**) obtained from the reversed monomeric library. Quantitative difference with models obtained from forward-oriented library likely comes from difference in sequences immediately flanking the TAANNN variable region. Figure 1—figure supplement 2—source data 1.

Intriguingly, several missense mutations within the same HD recognition helix as R90W, including p.E80A (E80A) and p.K88N (K88N), are linked to severe dominant IRDs (Freund et al., 1997; Swaroop et al., 1999; Nichols et al., 2010; Figure 1A, B). Here, we utilized a multi-omics approach to investigate the functional consequences of the E80A and K88N mutations on CRX regulatory activities and photoreceptor development (Figure 1—figure supplement 1). Comparison of the in vitro HD DNA-binding models of CRX and disease variants generated by Spec-seq revealed unique specificity changes of each mutant protein. Introduction of each mutation into the endogenous *Crx* locus generated knock-in mouse models *Crx^E80A^* and *Crx^K88N^* that reproduced dCoRD- and dLCA-like phenotypes. ChIP-seq analysis of CRX-binding in vivo revealed mutation-specific changes in CRX targetome, consistent with mutation-specific DNA-binding changes in vitro. Retinal RNA-seq analysis uncovered two distinct mechanisms by which the two HD missense mutations contribute to altered gene expression programs during photoreceptor differentiation and maturation. Our results highlight the importance of residues E80 and K88 in CRX-mediated transcriptional regulation during photoreceptor development and the diverse mechanisms by which HD missense mutations can affect TF functions and lead to severe dominant neurological diseases.

## Results

### K88N but not E80A mutation alters CRX HD DNA-binding specificity in vitro

CRX belongs to the paired-like HD TF family that recognize a 6-bp DNA motif in a stereotypic way (Desplan et al., 1988; Trelsman et al., 1989; Hanes and Brent, 1989; Hanes and Brent, 1991; Ades and Sauer, 1995; Wilson et al., 1995; Gehring et al., 1994). Extensive studies of the HD have revealed a canonical HD–DNA recognition model where recognition of the 3′ region (bases 4–6) of the HD DNA-binding site is mediated by specificity determinants within the conserved HD recognition helix, corresponding to CRX residues 80–96 (Desplan et al., 1988; Trelsman et al., 1989; Hanes and Brent, 1989; Hanes and Brent, 1991; Gehring et al., 1994; Noyes et al., 2008; Figure 1B). In particular, HD residue 50, equivalent to CRX K88 residue (Figure 1A), is the major specificity determinant in paired-like HD TF–DNA interactions (Trelsman et al., 1989; Hanes and Brent, 1989). Since the three disease-associated HD missense mutations, E80A, K88N, and R90W, are located within the CRX HD recognition helix, we wondered if these mutations change CRX HD DNA-binding specificity.

We adapted a high-throughput in vitro assay, Spec-seq, that determines protein–DNA-binding specificity by sequencing (Stormo et al., 2015; Zuo et al., 2017; Zuo and Stormo, 2014). Spec-seq was developed based on the traditional electrophoretic mobility shift assay (EMSA) to measure protein–DNA interactions. Spec-seq allows us to measure the relative binding affinities (i.e., specificity) for a library of HD-binding motifs in parallel and generate quantitative binding models for different CRX HDs (Figure 1C). Based on the HD–DNA interaction model, we designed and tested a Spec-seq library containing all possible monomeric HD motifs (TAANNN) (Figure 1D).

We first obtained the wild-type (WT) CRX HD DNA-binding model with Spec-seq using bacterially expressed and affinity-purified HD peptides (Figure 1E–G, Methods). Relative binding energies of CRX WT HD from two experiments showed strong correlation (*r*: 0.984) and noise level (0.114 kT) within the expected range in typical Spec-seq data (Figure 1F). Binding energy model of WT HD was then generated by applying multiple linear regressions on the relative binding energies of all sequences within two basepair mismatches to the WT CRX consensus (TAATCC) (Chen et al., 1997; Figure 1G, Methods). A clear preference for CC bases at the 3′ end of the motif is consistent with known CRX-binding preference in vitro and in vivo *(*Corbo et al., 2010; Kwasnieski et al., 2012).

We next sought to understand how disease mutations affect CRX DNA-binding specificity. We purified all mutant HD peptides following the same protocol as WT HD peptides and verified their DNA binding (Figure 1—figure supplement 2A-D; Chen et al., 2002). Comparison of the relative binding energies between each pair of mutant and WT HD revealed distinct effects (Figure 1H–J). By definition, the consensus DNA-binding motif of a testing peptide has a relative binding energy of 0 kT. The relative binding energy difference between nucleotide variants and the consensus motif correlates with the DNA-binding specificity of the testing peptide. We found that R90W HD and E80A HD both prefer the same consensus motif as WT (Figure 1K, L). R90W HD bound with slightly higher specificity than WT, as demonstrated by most data points falling above the identity line (Figure 1H, K), suggesting that R90W HD is more sensitive than WT to binding sequence variations. In contrast, when comparing E80A HD with WT HD, many data points fell below the identity line and the relative binding energies regressed toward 0 on the E80A axis (Figure 1I, L). This suggests that E80A HD bound with lower specificity than WT HD and thus was more tolerant to base variations in the HD DNA motif. Different from R90W and E80A, K88N mutation dramatically altered CRX HD DNA-binding specificity (*r*: 0.160) (Figure 1J, M). The K88N preferred binding sequence (TAAT/ATT/A) is referred to as N88 HD motif hereafter. K88N HD also had the largest degree of discrimination from its preferred to the weakest binding motif, suggesting that it is most sensitive to variants in the HD DNA motif. As a control, we tested a second library with the TAANNN sites on the reverse strand and obtained similar results (Figure 1—figure supplement 2E–L). Together, these results indicate that while E80A mutation does not affect CRX HD DNA-binding specificity, the K88N mutation dramatically alters the specificity in vitro.

### E80A protein binds to WT sites while K88N occupies novel genomic regions with N88 HD motifs in vivo

Next, we asked if changes in DNA-binding specificity affected mutant CRX chromatin binding in developing photoreceptors. We first created two human mutation knock-in mouse models, *Crx^E80A^* and *Crx^K88N^*, each carrying a single base substitution at the endogenous *Crx* locus (Figure 2—figure supplement 1A, B, Methods. For concision, we use *Crx^E80A^* and *Crx^K88N^* when both heterozygous and homozygous mutants are being discussed). We confirmed that *Crx* mRNA was expressed at comparable levels in WT and mutant retinas (Figure 2—figure supplement 1C), and the full-length CRX proteins were readily detectable in the nuclear extracts from all samples (Figure 2—figure supplement 1D). We then obtained genome-wide binding profiles for each CRX variant by chromatin immunoprecipitation followed by sequencing (ChIP-seq) on mouse retinas at P14, a time when all retinal cell types are born, photoreceptor specification is completed in WT animals, and prior to any observed cell death in other CRX mutants previously characterized (Tran et al., 2014; Bassett and Wallace, 2012). To focus on changes specific to each mutant CRX protein, only homozygous animals were used for ChIP-seq profiling.

**Figure 2.**
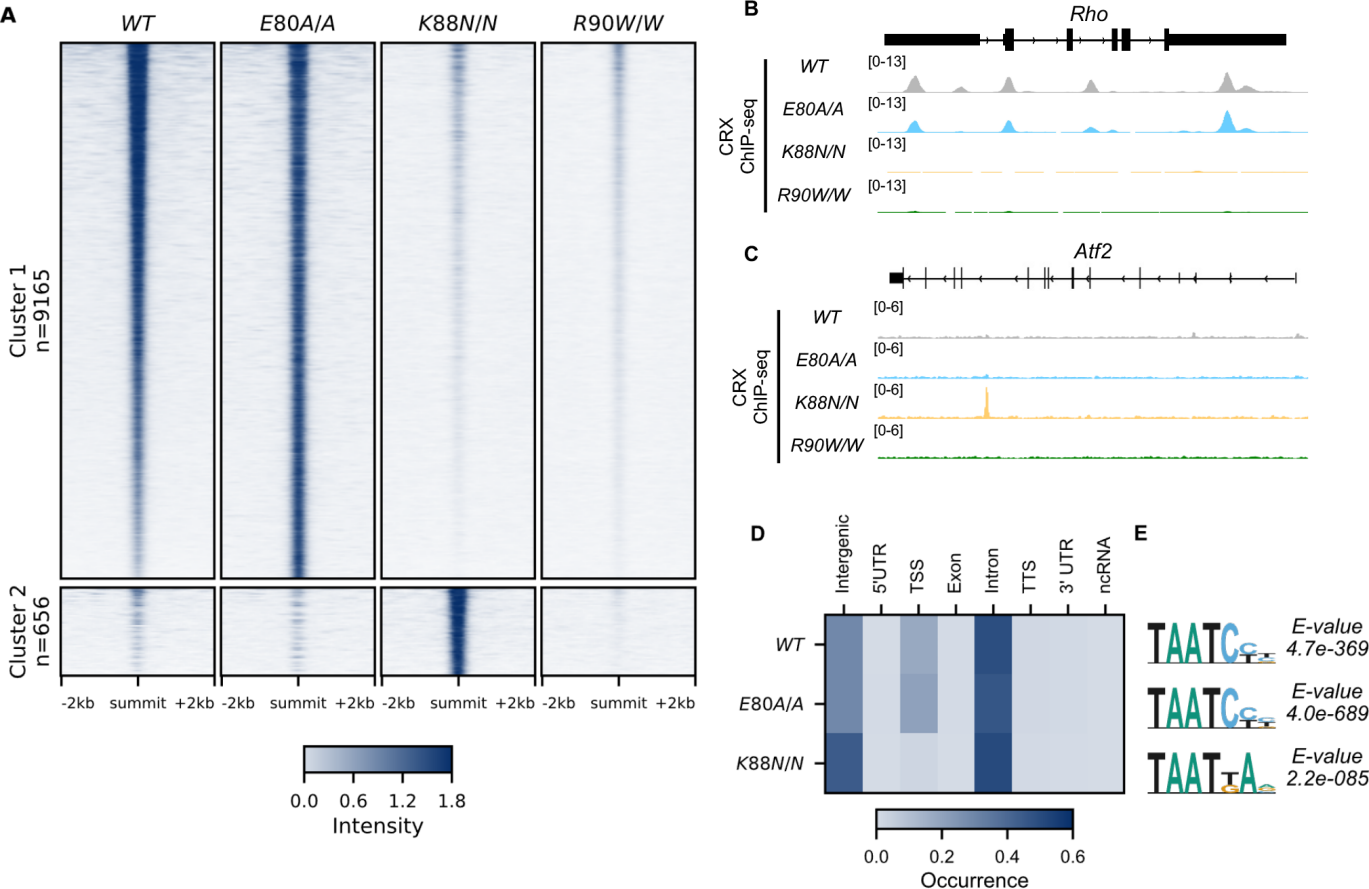
CRX E80A binds to wild-type (WT) sites while CRX K88N occupies novel genomic regions enriched for N88 homeodomain (HD) motif in vivo. (**A**) Enrichment heatmap depicting CRX ChIP-seq normalized reads centered at all possible CRX peaks ±2 kb, sorted by binding intensity in *WT* samples. Clusters were defined by hierarchical clustering of CRX-binding intensity matrix from all genotypes (Materials and methods). (**B, C**) Genome browser representations of ChIP-seq normalized reads for different CRX species in P14 WT and mutant mouse retinas at *Rho* and *Atf2*. (**D**) Enrichment heatmap showing fraction of CRX ChIP-seq peaks fall in different genomic environments. (**E**) Logo representations of de novo found short HD motifs under CRX ChIP-seq peaks in WT and mutant mouse retinas with DREME *E*-value on the right.

**Figure 3.**
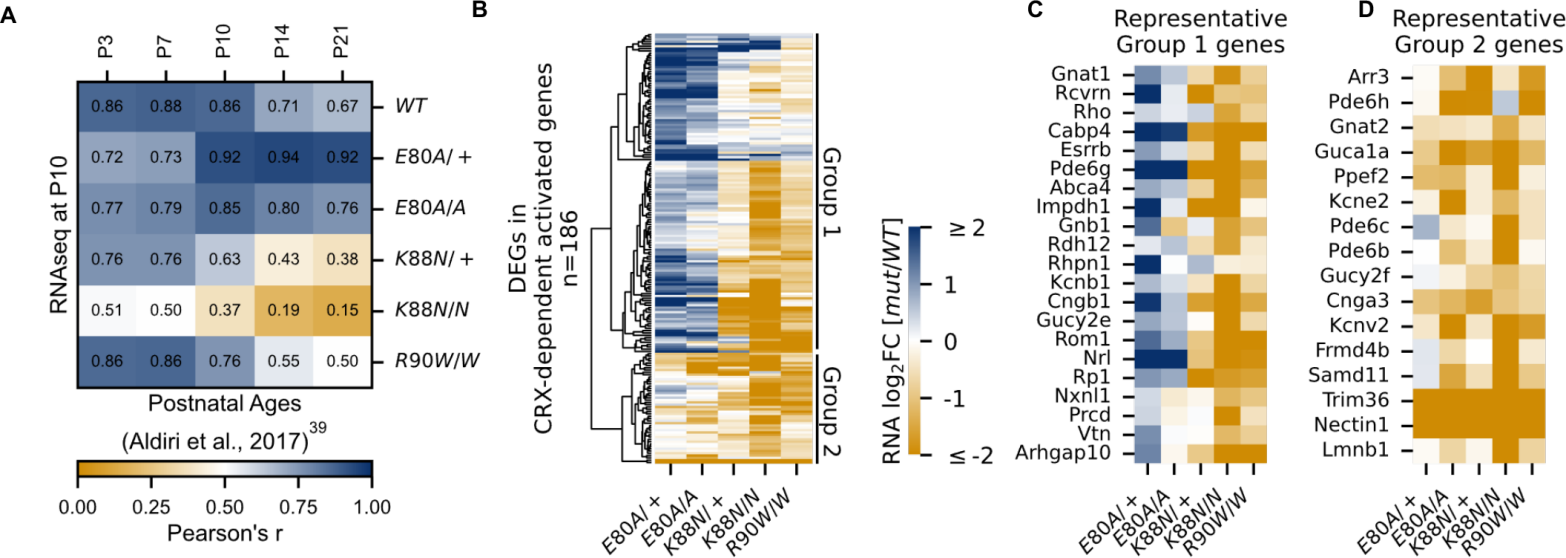
CRX-dependent activated genes affected in opposite directions in developing *Crx^E80A^* and *Crx^K88N^* mutant retinas. (**A**) Heatmap showing sample-wise Pearson correlations of the expression of all CRX-dependent activated genes between P10 wild-type (WT) and homeodomain (HD) mutant mouse retinas in this study (rows) with postnatal WT retinas from age P3 to P21 (columns, data from GSE87064). (**B**) Heatmap showing the expression changes of DEGs in CRX-dependent activated gene set in HD mutant mouse retinas at P10. (**C, D**) Heatmaps showing expression changes of selected photoreceptor genes from Groups 1 and 2. Color scale identical to (**B**).

Unsupervised clustering of all CRX-binding sites revealed two major clusters (Figure 2A, Methods). Cluster 1 consisted of canonical WT CRX-binding sites that are also occupied by CRX E80A protein (Figure 2A, B). Similar to WT CRX, CRX E80A-binding in vivo was mostly enriched in intronic, followed by intergenic and transcription start site (TSS) regions (Figure 3D). In contrast, CRX R90W, that also showed similar consensus preference to WT in vitro, failed to produce significant DNA binding in vivo (Figure 2A–C, Methods). This suggests that the retinopathy phenotype of *Crx^R90W/W^* is likely due to loss of binding at canonical WT CRX-binding sites. Intriguingly, while CRX K88N showed loss of binding at canonical CRX-binding sites, it gained a small set of binding sites (Cluster 2, Figure 2A–C). De novo motif searching with DREME (Bailey, 2011) under CRX peaks in each genotype revealed enrichment of monomeric HD motifs (Figure 2E) consistent with those found in Spec-seq (Figure 1G, K–M), highlighted by a change in enriched HD motif from WT CRX HD type to N88 HD type in the *Crx^K88N/N^* retinas. Consistency with in vitro binding models suggests that in vivo changes in CRX chromatin binding were at least in part driven by the intrinsic changes in HD DNA-binding specificity by each individual mutation.

**Figure 2—figure supplement 1.**
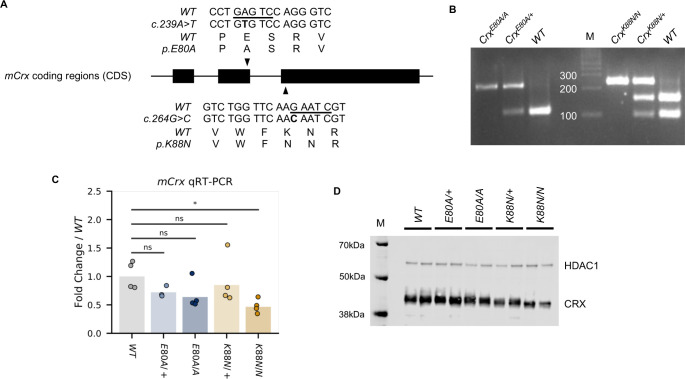
Wild-type (WT) and mutation knock-in mouse CRX sequences and genotyping identifications. (**A**) Alignment of *mCrx* cDNA and protein sequences showing the nucleotide substitutions and amino acid changes of *Crx^E80A^* (top) and *Crx^K88N^* (bottom) alleles. Only coding regions of *mCrx* exons are shown and the diagram is not to scale. Underlined bases in WT sequences indicate the restriction enzyme HinfI cut sites used in the genotyping PCR. (**B**) Representative mutation knock-in mouse genotyping gel image. (**C**) Barchart and stripplot showing *mCrx* mRNA expression levels in P14 WT and mutant mouse retinas. p-values for one-way analysis of variance (ANOVA) with Turkey honestly significant difference (HSD) test are indicated. (**D**) Immunoblots of nuclear extracts obtained from P14 WT and mutant mouse retinas showing that full-length CRX protein are produced and localized to the nucleus fraction in all mutant mouse retinas. HDAC1 was used as a loading control. Figure 2—figure supplement 1—source data 1.

**Figure 3—figure supplement 1.**
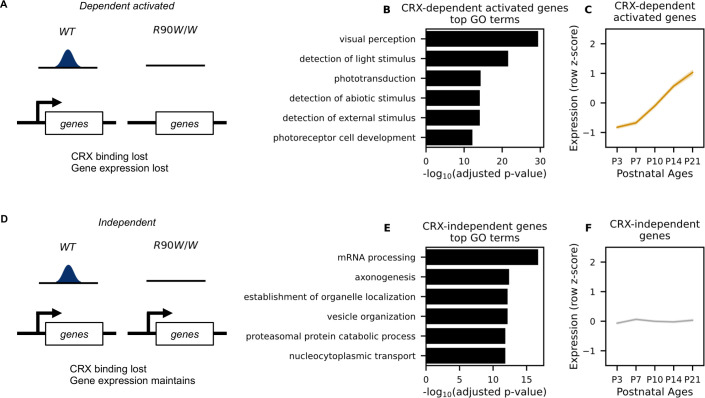
Definition of CRX-dependent activated and CRX-independent gene sets. (**A**) Schematic representation of CRX-dependent activated genes where CRX binding nearby is required for the expression of these genes in mature wild-type (WT) retinas. (**B**) Top gene ontology (GO) terms associated with CRX-dependent activated genes. Benjamini–Hochberg adjusted p-values are shown. (**C**) Line plot showing average expression pattern of CRX-dependent activated genes during normal postnatal retina. (**D**) Definition of CRX-independent genes where CRX binding nearby is dispensable for the expression of these genes in mature WT retinas. (**E**) Top GO terms associated with CRX-independent genes. (**F**) Line plot showing average expression pattern of CRX-independent genes during normal retina development. RNA-seq data in (**C**) and (**F**) were retrieved from Aldiri et al., 2017 (GEO accession: GSE87064).

**Figure 3—figure supplement 2.**
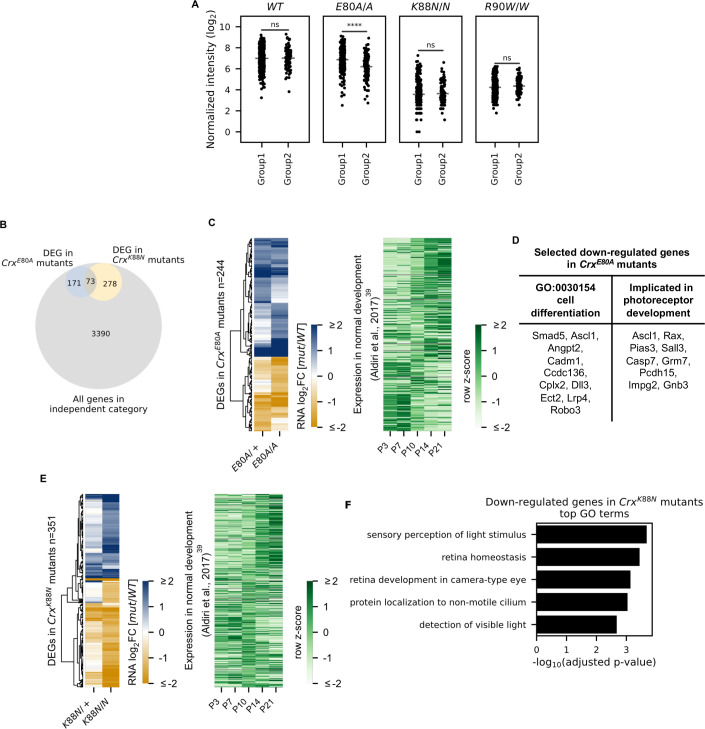
E80A and K88N mutation each causes novel gene expression changes in the CRX-independent category. (**A**) Strip plots showing normalized CRX ChIP-seq intensity at peaks associated with Group 1 or 2 genes in wild-type (WT) and CRX mutant mouse retinas. p-values for two-sided Mann–Whitney *U*-test are indicated. (**B**) Venn diagram showing the overlap of genes differentially expressed (DEGs) in *Crx^E80A^* (pale blue) and *Crx^K88N^* (pale yellow) but not in *Crx^R90W/W^* (gray) mutant retinas. For DEGs in *Crx^E80A^* and *Crx^K88N^* mutants, genes that were differentially expressed in either heterozygotes or homozygotes, or both were counted. (**C**) Heatmap showing the expression changes of CRX-independent genes that are DEGs in at least one of the *Crx^E80A^* mutants (*n* = 244, left). Heatmap on the right shows the expression pattern of these genes during normal postnatal development (data from GEO: GSE87064). (**D**) Table showing selected genes downregulated in *Crx^E80A^* mutants that have been implicated in cell differentiation or photoreceptor development. (**E**) Heatmap showing the expression changes of CRX-independent genes that are DEGs in at least one of the *Crx^K88N^* mutants (*n* = 351, left). Heatmap on the right shows the expression pattern of these genes during normal postnatal development (data from GEO: GSE87064). (**F**) Barchart showing gene ontology (GO) term enrichment of DEGs in *Crx^K88N^* mutants.

### E80A and K88N mutations affected the expression of CRX-dependent activated genes in opposite directions in a critical time window of photoreceptor differentiation

To understand how different CRX mutations affected gene expression at canonical and de novo binding sites and how these changes impair photoreceptor differentiation, we turned to bulk RNA-seq analysis from the developing retinas at P10. At P10, photoreceptors have started to differentiate, and the expression of many photoreceptor genes undergo exponential increase (Aldiri et al., 2017; Kim et al., 2016). To focus on the most relevant expression changes, we first defined a set of genes that most likely depend on CRX activity nearby for expression (Figure 3—figure supplement 1A, Methods). Briefly, we associated each CRX ChIP-seq peak to the nearest gene, filtered only genes with nearby CRX ChIP-seq peaks, and further narrowed the list of genes to those significantly downregulated in the loss-of-function mutant *Crx^R90W/W^*. Gene ontology (GO) analysis confirmed that this putative CRX-dependent gene set is associated with biological processes related to photoreceptor development and functions (Figure 3—figure supplement 1B). This set of putative CRX-dependent genes also showed developmental dependent gain in expression, consistent with CRX’s primary function as a transcriptional activator (Figure 3—figure supplement 1C). As a control, CRX-independent genes were constitutively expressed and largely involved in general cellular processes (Figure 3—figure supplement 1D–F). Therefore, the CRX-dependent gene set comprises genes important for photoreceptor differentiation and functional maturation and are dependent on CRX for activation. We denote these genes as ‘CRX-dependent activated genes’ (CRX-DAGs).

Next, we sought to understand how each mutation affected photoreceptor differentiation. One way of measuring the progression of photoreceptor differentiation is to determine the similarity in CRX-DAG expression in a given sample with that of known developmental ages in WT control animals. We thus performed sample-wise correlation of *CRX-DAG* expression obtained in our P10 samples with a previously published RNA-seq dataset of normal mouse retinal development (Figure 3A; Aldiri et al., 2017). As expected, our P10 WT sample showed strong correlations with all developmental ages in the published WT control dataset. A stronger correlation with early ages (P3, P7, P10) and a weaker correlation with later ages (P14, P21) is also an indication of ongoing photoreceptor differentiation at P10. Unlike the WT sample, *Crx^E80A/+^* and *Crx^E80A/A^* samples both showed a stronger correlation with later developmental ages (P14, P21) but a weaker correlation with earlier postnatal ages (P3, P7). Since the *CRX-DAGs* are normally developmentally upregulated, this shift in correlation toward later developmental ages suggested that these genes were prematurely upregulated in the P10 *Crx^E80A/+^* and *Crx^E80A/A^* mutant retinas. In contrast, *Crx^K88N/+^* and *Crx^K88N/N^* samples both showed a weaker correlation with all developmental ages when compared with WT samples in our dataset. This suggests that early photoreceptor differentiation was compromised in both *Crx^K88N^* mutants, consistent with their association with early-onset LCA (Nichols et al., 2010). Importantly, *Crx^R90W/W^*, also associated with LCA-like phenotype (Tran et al., 2014; Swaroop et al., 1999), displayed strong correlation with earlier ages (P3, P7) similar to WT, but only showed moderate correlation with later ages (P14, P21). This suggests loss of CRX function at canonical binding sites does not affect the initiation of photoreceptor differentiation, but WT CRX activity at these sites is required to sustain differentiation. Since *CRX-DAG* expression was more severely affected in *Crx^K88N^* mutants than in *Crx^R90W/W^*, the photoreceptor differentiation deficits seen in the *Crx^K88N^* mutants cannot be explained solely by the loss of regulatory activity at canonical CRX-binding sites. Overall, our sample-wise correlation analysis with normal retinal development dataset suggests that E80A and K88N mutations affected the expression CRX-dependent activated genes in opposite directions, implicating novel and distinct pathogenic mechanisms from the loss-of-function R90W mutation.

### *Crx^E80A^* retinas show upregulation of rod genes but downregulation of cone genes, underlying CoRD-like phenotype

Upon closer examination, we noted that not all *CRX-DAGs* were upregulated in *Crx^E80A^* mutants (Figure 3B). Hierarchical clustering of all *CRX-DAGs* using expression changes revealed two major groups (Methods). In aggregate, when compared to WT, Group 1 genes were upregulated in *Crx^E80A^* mutants while Group 2 genes were downregulated. We noted genes indicative of the two photoreceptor subtypes, rods and cones, could partially define the two groups (Figure 3C, D). For example, *Esrrb* (Onishi et al., 2010) and *Nrl* (Yoshida et al., 2004) in Group 1 are important regulators of rod differentiation. Other genes in Group 1 are components of the phototransduction cascade in rods, including *Rcvrn (*Zang and Neuhauss, 2018), *Rho (*Palczewski, 2006), *Gnat1 (*Dryja et al., 1996; Carrigan et al., 2016), *Pde6g (*Dvir et al., 2010), *Abca4 (*Allikmets et al., 1997; Nasonkin et al., 1998), *Gnb1 (*Kitamura et al., 2006), *Rdh12 (*Janecke et al., 2004), *Cngb1 (*Bareil et al., 2001), and *Rp1* (Pierce et al., 1999; Sullivan et al., 1999). Mis-regulations of many of these genes have been associated with diseases that affect rod development, function, and long-term survival. The increased activation of these genes likely underlies the stronger correlation with later developmental ages in *Crx^E80A^* mutant retinas (Figure 3A). In contrast, Group 2 genes, many downregulated in *Crx^E80A^* mutants, were implicated in cone development and functions. For example, *Gnat2 (*Kohl et al., 2002; Rosenberg et al., 2004), *Pde6c* (Thiadens et al., 2009), and *Pde6h (*Kohl et al., 2012; Piri et al., 2005) all act in the cone phototransduction cascade. Mis-regulation of these genes has also been implicated in different retinal dystrophies that primarily affect cone photoreceptors. Comparison of ChIP-seq signal revealed that peaks associated with Group 2 genes showed lower occupancy compared to Group 1 genes in the *Crx^E80A/A^* retinas (Mann–Whitney *U*-test p-value: 9.51e−07) while no difference was observed in WT retinas (Mann–Whitney *U*-test p-value: 0.541), suggesting loss of CRX activity likely underlies the downregulation of Group 2 genes in the *Crx^E80A^* mutants (Figure 3—figure supplement 2A). Collectively, the selective downregulation of cone genes in Group 2 may explain the CoRD-like phenotype in adult *Crx^E80A^* mutant mice described later.

Additionally, we noticed that a subset of genes not affected in *Crx^R90W/W^* (CRX-independent genes) were also downregulated in *Crx^E80A^* mutants (Figure 3—figure supplement 2B–D). Among these genes were transcription regulators important for early photoreceptor development, such as *Ascl1* (Kaufman et al., 2019), *Rax* (Irie et al., 2015), *Sall3* (de Melo et al., 2011), and *Pias3* (Onishi et al., 2009). The downregulation of these factors coincided with the upregulation of mature rod genes in P10 *Crx^E80A^* retinas, suggesting that the E80A mutation might hamper the proper timing of photoreceptor differentiation.

### *Crx^K88N^* retinas display greater reduction of rod and cone genes than the loss-of-function mutants

*Crx^K88N^* retinas had the most severe gene expression changes among all mutants with downregulation of both Group 1 (rod) and Group 2 (cone) genes (Figure 3B–D). The heterozygous *Crx^K88N/+^* retina displayed a similar degree of expression reduction as homozygous *Crx^R90W/W^*, consistent with its association with dLCA (Nichols et al., 2010). Given the normal phenotype of heterozygous loss-of-function mutants – *Crx^+/−^* and *Crx^R90W/+^*, these results suggest that mutant CRX K88N not only failed to activate WT target genes, but also functionally antagonized WT CRX regulatory activity in differentiating photoreceptors (Tran et al., 2014). This antagonism might be associated with ectopic CRX K88N activity when bound to regulatory regions with N88 HD DNA motifs (+/−). In the absence of WT CRX, *Crx^K88N/N^* retina displayed a more severe expression reduction of *CRX-DAG* than *Crx^R90W/W^*, raising the possibility that CRX K88N also antagonized the activity of other transcriptional regulators important for photoreceptor differentiation. Supporting this possibility, a set of CRX-independent genes were also mis-regulated in both heterozygous and homozygous *Crx^K88N^* mutants. We noted that a number of these downregulated genes are also involved in photoreceptor functional development (Figure 3—figure supplement 2B, E, F). Overall, CRX K88N is associated with greater gene expression changes than other CRX HD mutants, which is likely attributed to ectopic regulatory activity.

### Both *E80A* and *K88N* mutants show compromised rod/cone terminal differentiation in young adults

**Figure 4.**
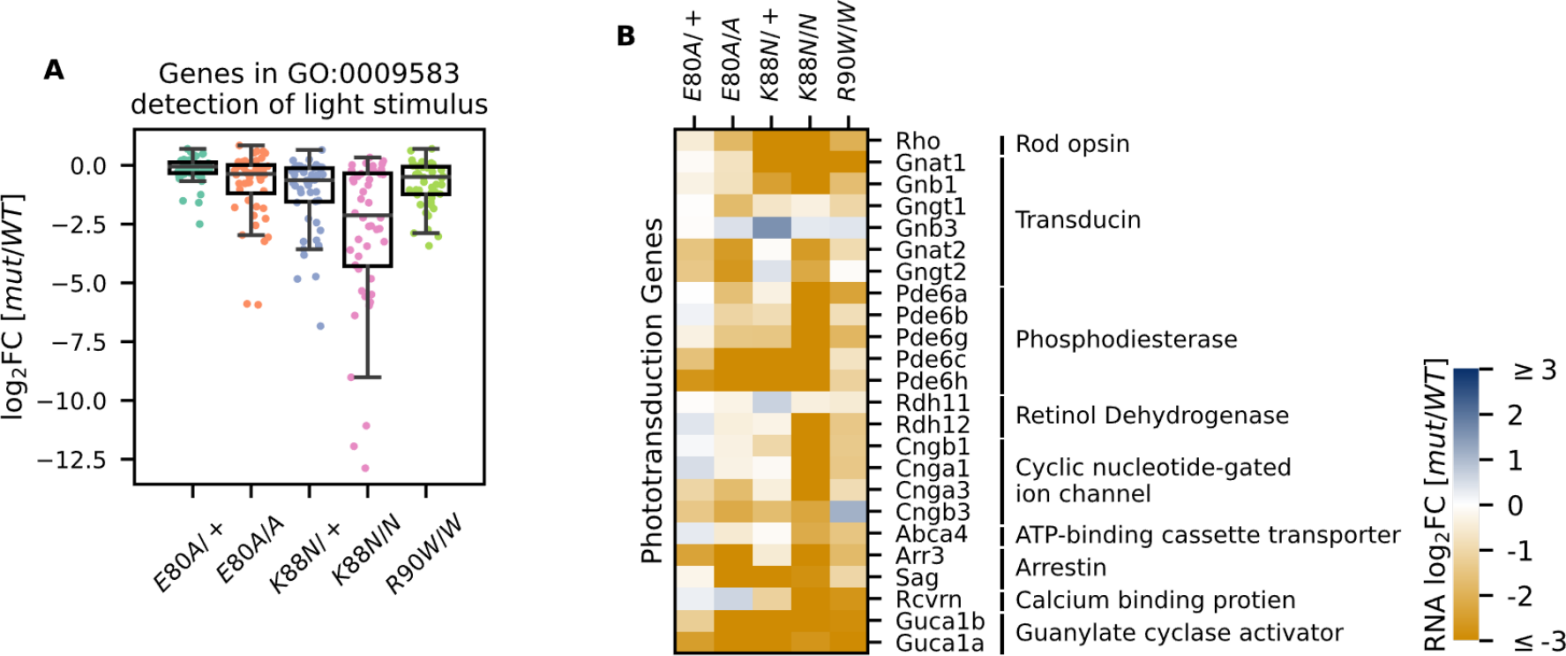
Photoreceptor genes important for phototransduction are downregulated in all homeodomain (HD) mutants. (**A**) Box plot showing that genes in the detection of light stimulus gene ontology (GO) term were downregulated and affected to various degrees in different adult (P21) HD mutant mouse retinas. (**B**) Heatmap showing that expression of both cone and rod phototransduction genes were downregulated in adult (P21) HD mutant mouse retinas. Annotation of rod and cone enrichment of each gene is in Supplementary file 1f. See Figure 4—figure supplement 1 for the developmental expression dynamics of these genes.

Since *Crx^+/−^* and *Crx^R90W/+^* mutant mouse models showed a late-time recovery in photoreceptor gene expression and function (Ruzycki et al., 2015), we sought to determine the degree of photoreceptor differentiation in *Crx^E80A^* and *Crx^K88N^* mutants at P21. At this age, the normal retina has largely completed terminal differentiation with photoreceptor gene expression reaching a plateau (Aavani et al., 2017). However, when P21 CRX HD mutant retinas were examined for the expression of genes under the GO term detection of light stimulus (GO:0009583), which comprises genes in both rod and cone phototransduction cascades, many genes failed to reach WT levels, despite variable degrees of impact across different HD mutants (Figure 4A). *Crx^E80A^* mutants, in contrast to the increased rod gene expression at P10, displayed a deficiency in both cone and rod phototransduction genes at P21 (Figure 4B, Supplementary file 1h). This suggests that mutant CRX E80A transcriptional activity fails to sustain photoreceptor terminal differentiation and ultimately results in non-functional and severely affected photoreceptors. In comparison, *Crx^K88N^* mutants showed severely reduced expression of rod/cone phototransduction genes at both P10 and P21 (Figures 3B–D–4B).

**Figure 4—figure supplement 1.**
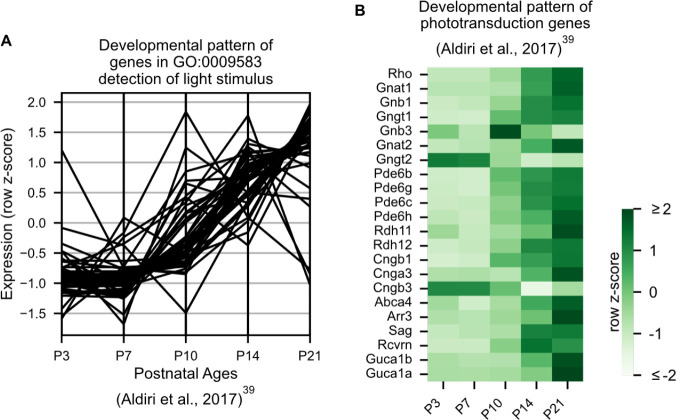
Developmental expression pattern of phototransduction genes in *WT* animals. (**A**) Parallel coordinates plot showing expression pattern of genes in GO:0009583 during normal postnatal retina development (data from GEO: GSE87064). Row *z*-score is shown. (**B**) Heatmap showing expression patterns of phototransduction genes during normal postnatal retina development (data from GEO: GSE87064). Row *z*-score is shown.

**Figure 5.**
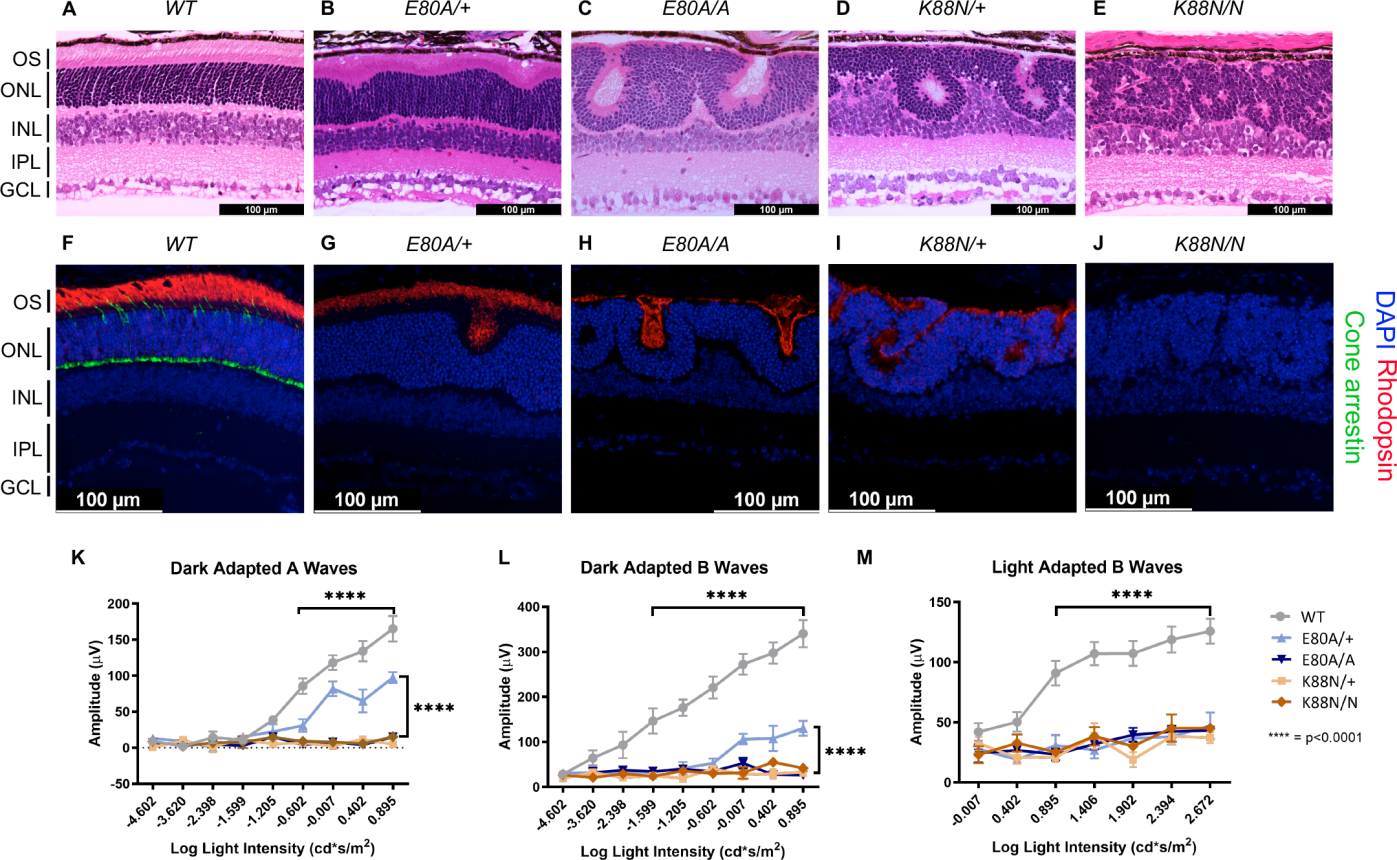
Only *Crx^E80A^*^/+^ retinas maintain photoreceptor OS and residual rod electroretinogram (ERG) response. (**A–E**) Hematoxylin–eosin (H&E) staining of P21 retina sections show that photoreceptor OS layer is absent in all mutant retinas except *Crx^E80A/+^*. OS: outer segment; ONL: outer nuclear layer; INL: inner nuclear layer; IPL: inner plexiform layer; GCL: ganglion cell layer. Scale bar, 100 µm. (**F–J**) Rhodopsin (RHO, red) immunostaining is present in *Crx^E80A/+^*, *Crx^E80A/A^*, and *Crx^K88N/+^* retinas and absent in *Crx^K88N/N^* retina. Cone arrestin (mCAR, green) immunostaining is absent in all mutant retinas. Nuclei were visualized by (4',6-diamidino-2-phenylindole) DAPI staining (blue). Scale bar, 100 µm. (**K–M**) The ERG responses recorded from 1 month mice. Error bars represent the standard error of the mean (SEM, *n* ≥ 4). p-value: Two-way analysis of variance (ANOVA) and Tukey’s multiple comparisons. ****p ≤ 0.0001. ns: >0.05.

To assess retinal morphology and photoreceptor subtype-specific gene expression at the cellular level, we performed immunohistochemistry analysis on P21 retinal sections. In WT animals, a hallmark of photoreceptor maturation is the outgrowth of photoreceptor outer segments (OS) filled with proteins necessary for the phototransduction. We thus performed hematoxylin and eosin (H&E) staining on P21 sagittal retinal sections to visualize changes in retinal layer organization, focusing on photoreceptor layers – outer nuclear layer (ONL) and OS. Compared to the well-organized ONL in WT retinas (Figure 5A), all mutants showed variable degrees of ONL disorganization, forming waves, whorls, and rosettes (Figure 5B–E). The ONL disorganization was more severe in homozygotes than in heterozygotes for both mutations and *Crx^K88N^* mutants were more severely affected than *Crx^E80A^* mutants. Photoreceptor OS layer was formed in the *Crx^E80A/+^* retinas, but absent in *Crx^E80A/A^*, *Crx^K88N/+^*, and *Crx^K88N/N^* mutant retinas. Inner retinal layers, including inner plexiform layer (IPL) and ganglion cell layer were not as severely affected as the outer retinal layers, supporting a model that the mutant morphological abnormalities largely originated from the diseased photoreceptors. These morphological abnormalities were distinct from the degenerative phenotypes of other *Crx* mutant models reported previously (Tran et al., 2014; Tran and Chen, 2014; Roger et al., 2014).

Next, we sought to determine the expression of the rod-specific visual pigment rhodopsin (RHO) and cone arrestin (mCAR) in the P21 mouse retinas. In WT retinas, RHO is trafficked to the rod OS while mCAR is present in the cone OS and IS (inner segment), cell body, and synaptic terminals (Figure 5F). Unlike WT retina, all mutants lacked mCAR immunoreactivity (Figure 5G–J), consistent with the loss of cone gene expression shown by RNAseq (Figure 4B). In *Crx^E80A/+^* retinas, RHO staining was localized to the OS layer; in *Crx^E80A/A^* and *Crx^K88N/+^* retinas, positive RHO staining was observed within the whorls and rosettes; in *Crx^K88N/N^* mutant retinas, RHO staining was completely absent. Importantly, we did not observe mis-localized RHO staining in the inner retinal layers (INL) suggesting that the developmental programs of other retinal cell types were not directly affected by E80A or K88N mutation. Overall, abnormalities in the cone/rod gene expression matched the corresponding human disease diagnosis (Freund et al., 1997; Nichols et al., 2010), and the phenotypic severity correlated with the degree of mis-regulation of CRX target genes in the corresponding RNAseq dataset. Thus, these results support a model that CRX HD mutation-mediated mis-regulation of gene expression disrupts photoreceptor terminal differentiation and leads to defects in retinal layer organization and OS formation.

### *E80A* and *K88N* mouse models show visual function deficits that recapitulate human diseases

To understand the consequences of disrupted photoreceptor differentiation on visual function, we measured electroretinogram (ERG) responses to light stimuli for WT and mutant mice at 1 month of age (Figure 5K–M). In response to incremental changes of light intensities, WT animals showed corresponding amplitude increases in dark-adapted A-waves (rod signals) and B-waves (rod-evoked bipolar cell signals), as well as in light-adapted B-waves (cone-evoked bipolar cell signals). The three severe mutants, *Crx^E80A/A^*, *Crx^K88N/+^*, and *Crx^K88N/N^* had no detectable dark- or light-adapted ERG responses, suggesting that these mice have no rod or cone function and are blind at young ages. The null ERG phenotype of the *Crx^K88N^* animals is consistent with the clinical LCA phenotype in humans (Nichols et al., 2010). In contrast, *Crx^E80A/+^* animals retained partial rod ERG responses as indicated by the reduced A- and B-wave amplitudes (Figure 5K, L). Yet, *Crx^E80A/+^* animals had no detectable cone ERG responses, which is consistent with the CoRD clinical phenotype in humans (Freund et al., 1997; Figure 5M). Taken together, the visual function impairment in each CRX HD mutant model, coincided with the morphological and molecular changes, suggesting that *Crx^E80A^* and *Crx^K88N^* mouse models recapitulate the corresponding human diseases.

### CRX E80A has increased transactivation activity and leads to precocious differentiation in *Crx^E80A^* retinas

**Figure 6.**
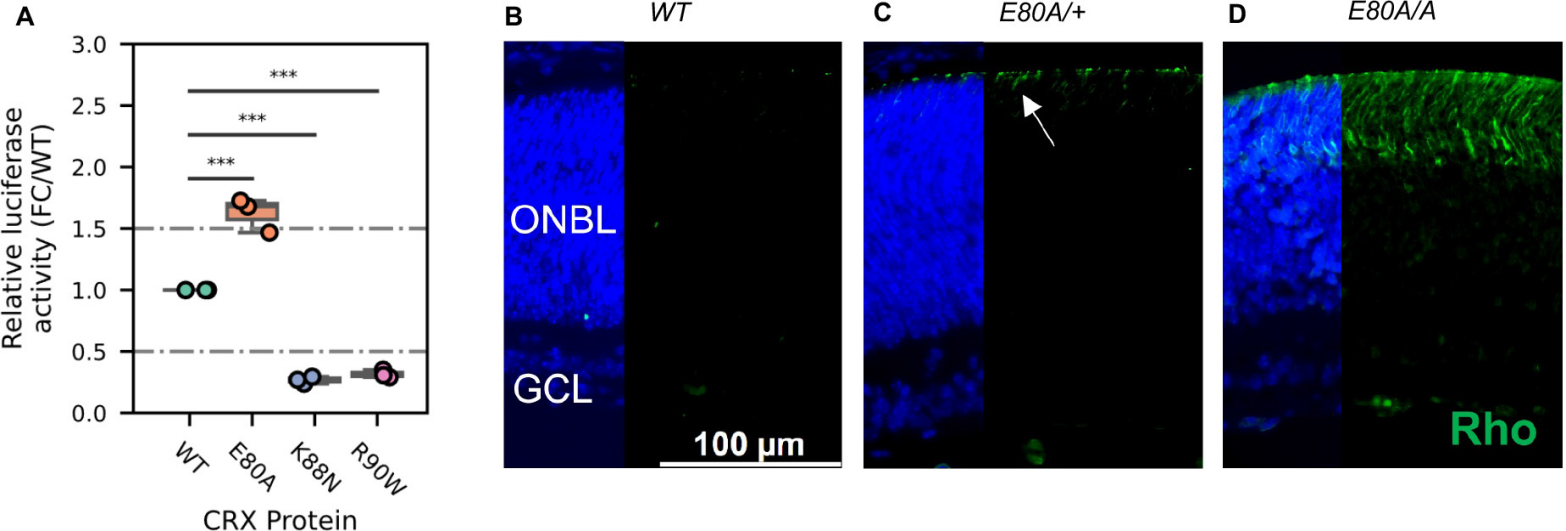
CRX E80A hyperactivity underlies precocious photoreceptor differentiation in *Crx^E80A^* retinas. (**A**) Boxplot showing luciferase reporter activities of different CRX variants. p-values for one-way analysis of variance (ANOVA) with Turkey honestly significant difference (HSD) test are indicated. (**B–D**) Rhodopsin (RHO, green) immunostaining is absent in P3 wild-type (WT) retina but detected in *Crx^E80A/+^* and *Crx^E80A/A^* retinas. Nuclei are visualized by DAPI staining (blue). Arrow indicates the sporadic RHO staining in *Crx^E80A/+^* sample. ONBL: outer neuroblast layer; GCL: ganglion cell layer. Scale bar, 100 µm.

Lastly, we asked what might be the molecular mechanism that causes the mis-regulation of photoreceptor genes in the mutant retinas. Previous studies have established that reporter assays with the *Rhodopsin* promoter in HEK293T cells can measure changes of CRX transactivation activity and inform the mechanisms by which photoreceptor genes are mis-regulated in CRX mutant retinas (Chen et al., 2002). We thus tested the transactivation activity of the three CRX HD mutants on the pRho-Luc reporter in HEK293T cells (Figure 6A, Methods). Consistent with published studies, R90W mutant had significantly reduced activity compared to WT CRX (Chen et al., 2002). K88N mutant showed a similarly reduced activity as R90W, consistent with K88N’s loss of binding at canonical CRX sites and failure to activate *CRX-DAGs* in vivo (Figures 2A, B, 3A–C). In contrast, E80A mutant, which binds to canonical CRX sties, showed significantly increased transactivation activity on the *Rho* promoter. This hyperactivity of E80A protein at *Rho* promoter correlates with the upregulation of *CRX-DAGs* in the mutant retinas at P10 (Figure 3A–C).

**Figure 6—figure supplement 1.**
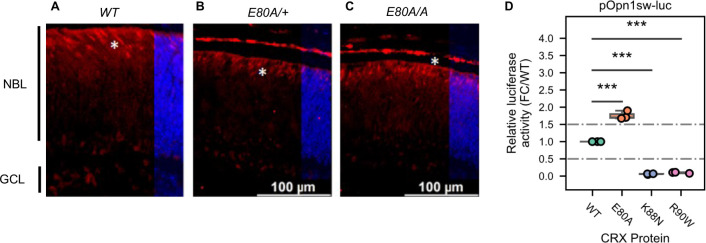
Cone photoreceptors born in *Crx^E80A^* retinas and hyperactivity of CRX E80A at S-opsin promoter. (**A–C**) Immunostaining shows that Retinoid X receptor gamma (RXRγ, red), a fated cone photoreceptor marker, is present in P0 *WT*, *Crx^E80A/+^*, and *Crx^E80A/A^* retinas. Nuclei are visualized by DAPI staining (blue). Asterisks indicate examples of RXRG+ cells. NBL: neuroblast layer; GCL: ganglion cell layer. Scale bar, 100 µm. (**D**) Boxplot showing luciferase reporter activities of different CRX variants at the *Opn1sw* promoter sequences. p-values for one-way analysis of variance (ANOVA) with Turkey honestly significant difference (HSD) test are indicated.

A transition to the next developmental stage usually requires the expression of important developmental genes passing an abundance threshold. Based on the hyperactivity model, photoreceptor genes are activated stronger in *Crx^E80A^* retinas and thus could reach the abundance threshold earlier. To determine the consequences of E80A hyperactivity on photoreceptor differentiation timing, we compared RHO protein expression during early postnatal retinal development using retinal section immunostaining (Figure 6B–D). In WT retinas, most rods were born by P3 but had not differentiated (Figure 6B). Previous studies showed that RHO proteins were detected by IHC starting around P7 in WT retinas (Aavani et al., 2017). In comparison, both *Crx^E80A/+^* and *Crx^E80A/A^* retinas showed positive RHO staining at P3 (Figure 6C, D). RHO^+^ cells were largely seen in the outer portion of the ONBL layers in *Crx^E80A/+^* retinas, and strikingly spread throughout the large presumptive ONL layers in *Crx^E80A/A^* retinas. The detection of RHO protein in P3 *Crx^E80A^* mutant retinas indicates that photoreceptor differentiation program was precociously activated. Taken together, our results support a model that *E80A* and *K88N* mutations each perturbs CRX regulatory activity in a unique way, causes photoreceptor differentiation defects, and ultimately leads to distinct dominant disease phenotype that recapitulates human diseases.

## Discussion

**Figure 7.**
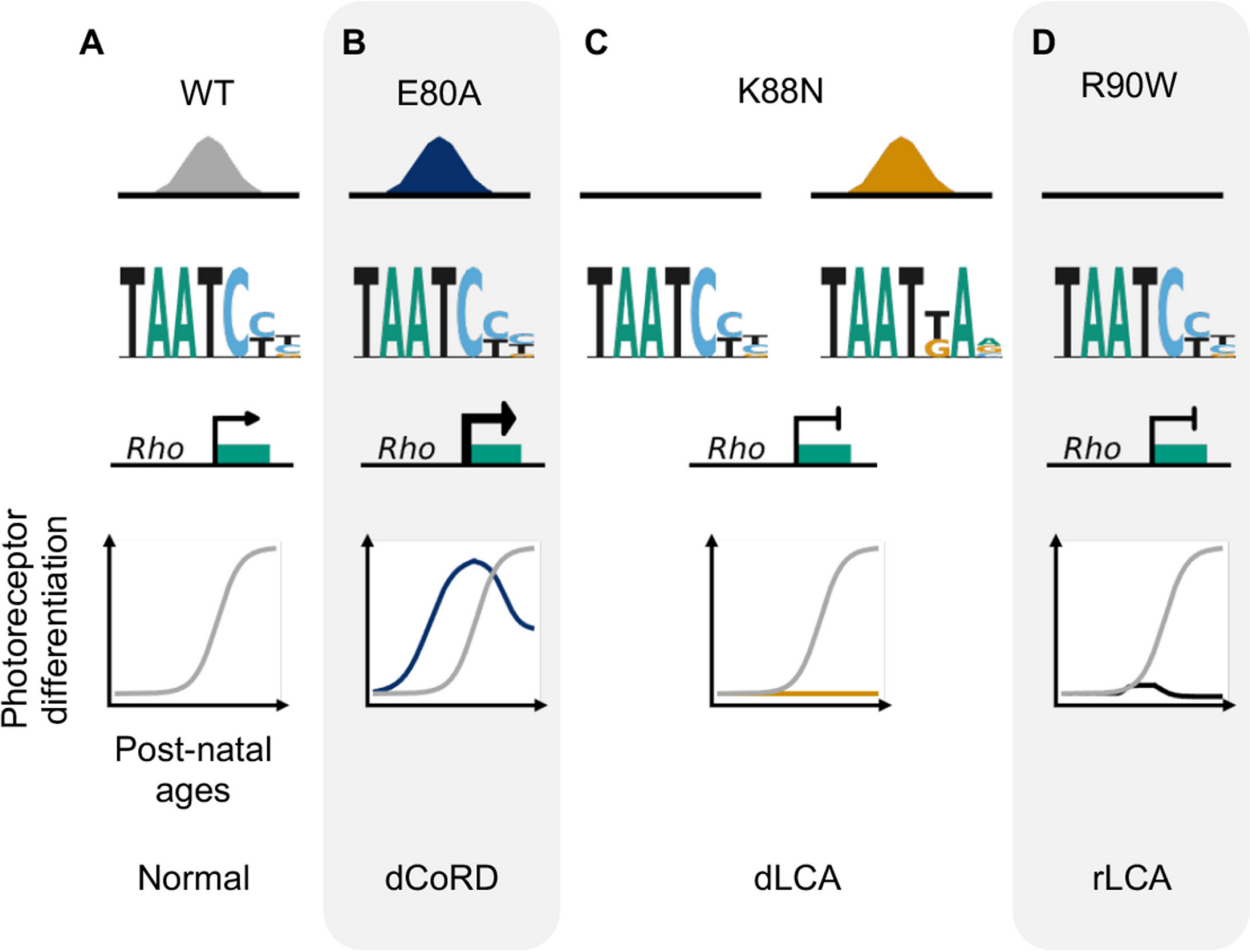
Missense mutations in CRX homeodomain (HD) affect photoreceptor gene expression and lead to distinct retinal disease phenotypes through gain- and loss-of-function mechanisms. dCoRD: dominant cone–rod dystrophy; dLCA/rLCA: dominant/recessive Leber congenital amaurosis.

Through molecular characterization of mutant proteins, transcriptome, and cellular profiling of developing mutant mouse retinas and ERG testing of adult retinas, we have identified two novel pathogenic mechanisms of CRX HD mutations, E80A and K88N, that are associated with dominant CoRD and dominant LCA in human (Freund et al., 1997; Swaroop et al., 1999). Distinct from the previously characterized loss-of-function R90W mutation (Tran et al., 2014; Ruzycki et al., 2015), E80A and K88N mutations produce altered CRX proteins with gain of regulatory functions – CRX E80A is associated with increased transcriptional activity and CRX K88N has altered DNA-binding specificity. Both CRX E80A and CRX K88N proteins impair photoreceptor gene expression, development and produce structural and functional deficits in knock-in mouse models, recapitulating human diseases (Tran and Chen, 2014; Figure 7). Thus, both target specificity and regulatory activity precision at the canonical CRX targets are essential for proper photoreceptor development and functional maturation.

Although associated with distinct disease phenotypes, the E80A, K88N, and R90W mutations are located very close to each other in the CRX HD recognition helix. Extensive biochemical and structural studies on the HD–DNA complexes afford important insights into how these mutations could affect CRX HD–DNA interactions differently. Most distinctively, CRX K88 residue, at HD50 position, is the major contributor to HD DNA-binding specificity, with lysine making favorable interactions with both guanines in the CRX consensus TAATCC-binding site (Baird-Titus et al., 2006; Chaney et al., 2005). It is thus expected that K88N mutation drastically changes CRX DNA-binding preference at the 3′ end of the HD motif, reminiscent of previous findings on novel HD DNA-binding specificity using bacterial one-hybrid (B1H) systems (Noyes et al., 2008; Chu et al., 2012). Supporting the importance of K88 residue-mediated CRX target specificity in regulating photoreceptor development, *Crx^K88N^* retinas show more severe perturbations in photoreceptor gene expression and development than in loss-of-function mutant *Crx^R90W/W^. Crx^K88N/+^* and *Crx^K88N/N^* mice display the most severe photoreceptor morphological deficits observed in any *Crx* mouse models and show absence of visual functions in young adults. Thus, CRX target specificity is critical for photoreceptor development fidelity.

In developing WT mouse retinas, the HD motif preferred by HD TFs with a glutamine (Q) at HD50 position encodes quantitatively different activity than the CRX consensus suggesting functional difference between HD-binding site variants (Irie et al., 2015; Kimura et al., 2000). It is likely that the severe *Crx^K88N^* phenotypes are attributed to both diminished activity at canonical CRX motifs and ectopic binding and transcriptional activity at N88 HD motifs. Since a functional copy of WT CRX is retained in *Crx^K88N/+^* retinas, the lack of WT activity alone cannot explain the severe developmental deficits. Alternatively, these results suggest involvement of additional regulatory mechanisms: CRX K88N activity at N88 HD motifs might (1) ectopically activate genes whose expression prevents the progression of development or inactivate genes required for development; (2) interfere with other HD TFs that also recognize N88 HD motifs; (3) lead to epigenetic alterations that antagonize normal CRX functions. Many other HD containing TFs are expressed in developing mouse retina, including OTX2, RAX, VSX2, PAX6, SIX3/6, and LHX family (Diacou et al., 2022). Different from CRX, these HD TFs are essential for gene regulation in retinal progenitor cells and/or in other retinal cell lineages. Alteration of CRX DNA-binding specificity could mis-regulate genes originally targeted by these HD TFs and lead to severe perturbations in the retinal gene regulatory networks. To date, most studies have focused on CRX activity at cis-regulatory sequences enriched for the WT CRX consensus motifs (Hughes et al., 2018; White et al., 2016). Systematic comparison of regulatory activity at N88 HD motifs and WT consensus in the context of photoreceptor development in both WT and mutant retinas would be needed to substantiate the impact of mutant CRX K88N activity at different HD motifs. These experiments will also help clarify the pathogenic mechanisms in the *Crx^K88N^* models and extend our knowledge of CRX HD-mediated regulatory grammar during photoreceptor development.

Different from CRX K88, the E80 and R90 residues, although the most common residues at HD42 and HD52 positions, respectively, do not contact DNA directly and thus lacked in-depth investigations in prior studies. CRX R90 residue has been suggested to confer additional stability for the HD fold besides the core residues and make contacts with the DNA backbone through bases in the TAAT core motif (Baird-Titus et al., 2006; Chaney et al., 2005). Substitution of the basic R90 residue with a bulky, neutral tryptophan (W) potentially reduces overall CRX HD stability which in term reduces CRX HD DNA-binding affinity without affecting its binding preference. The potential reduction in CRX HD–DNA complex stability is in line with our observation that R90W mutation abolishes CRX binding across the genome resulting in global loss of CRX target gene activation. It also explains the association of CRX R90W mutation with recessive loss-of-function LCA phenotypes in human and mouse (Swaroop et al., 1999; Tran and Chen, 2014).

Structural studies suggest that CRX E80 residue plays a role in stabilizing the HD–DNA-binding complex through intramolecular interactions with other HD residues (Wilson et al., 1995; Chaney et al., 2005), yet functional validations await further experiments. E80A mutation, replacing glutamic acid (E), which is acidic and polar, with alanine (A), which is neutral and non-polar, could render HD–DNA interactions more promiscuous as reflected in overall reduced magnitude of CRX E80A HD specificity (Figure 1I). Regulatory sequences of many photoreceptor genes contain both consensus and non-consensus CRX motifs (Chen et al., 1997; Furukawa et al., 1997; Chen et al., 2002; Corbo et al., 2010). It is likely that the more promiscuous CRX E80A–DNA interaction increases the likelihood of non-consensus CRX HD motifs being bound and activated resulting in overall increased transcriptional output (hyperactivity) as seen both in luciferase assays and in developing *Crx^E80A^* mouse retinas. The promiscuous TF-DNA-binding associated hyperactivity phenomenon has also been observed in a dominant disease mouse model harboring a missense mutation in the zinc finger TF Krüppel-like factor-1 (KLF1) (Gillinder et al., 2017). Yet, in adult *Crx^E80A^* retinas, photoreceptor terminal differentiation is impaired, resulting in disrupted retinal morphology and defective visual functions. Photoreceptor differentiation is programmed via sequential and concerted gene expression programs within a defined time window (Wang and Cepko, 2016; Brzezinski and Reh, 2015). One explanation for adult *Crx^E80A^* phenotypes is that CRX E80A hyperactivity precociously activates later stage genes in the absence of proper nuclear context and/or subcellular structures, which in turn negatively impacts early events in photoreceptor differentiation. These observations underscore the importance of precisely tuned CRX-mediated transcriptional activity during photoreceptor development.

Although a global increase in expression is expected in *Crx^E80A^* retinas based on the hyperactivity model, a subset of CRX-dependent activated genes implicated in cone photoreceptor development and functions is downregulated in both differentiating and mature mutant retinas. While cones undergo terminal differentiation to develop into cone subtypes – M- or S-cones – in a similar postnatal window as rods, they were born prenatally in mice within an earlier time window than rods. At an early postnatal age, cells expressing RXRγ, a ligand-dependent nuclear hormone receptor normally expressed in developing cones (Kaufman et al., 2019; Sapkota et al., 2014; Mori et al., 2001; Roberts et al., 2005), were observed in *Crx^E80A^* retinas, suggesting cone photoreceptors were born in these mutant retinas (Figure 6—figure supplement 1A). One model for lack of cone markers in adult *Crx^E80A^* retinas is that CRX E80A improperly activates later stage cone genes at a much earlier time window, disrupting cone terminal differentiation. Supporting this model, CRX E80A also hyperactivates the S-cone opsin promoter reporter pOpn1sw-luc (Figure 6—figure supplement 1B). An alternative model is that cones might be more sensitive to perturbations in CRX activity. It is known that cones depend on a different repertoire of TFs than rods for subtype terminal differentiation (Kaufman et al., 2019). It is possible that cone TFs respond differently to mutant CRX E80A hyperactivity, leading to the distinct expression changes in *Crx^E80A^* mutant retains. It is important to note that different point mutations at CRX E80 residue have been reported in dominant CoRD cases (ClinVar VCV000865803.1, VCV000007416.7, VCV000099599.6), emphasizing the importance of residue CRX E80 in regulating cone photoreceptor development. Since cones only make up a very small portion (3%) of photoreceptors in mouse retinas (Carter-Dawson and LaVail, 1979), quantitative characterization of CRX E80A molecular functions in a cone dominant retina warrants further study to understand its selective effect on the cone differentiation program and help elucidate WT CRX regulatory principles in early photoreceptor development.

Given that the spatial structures and HD–DNA contact models of HD proteins are evolutionarily conserved, our study of CRX provides valuable molecular insights for HD mutations implicated in other diseases. For example, p.E79K substitution (corresponds to CRX E80) in OTX2 HD is associated with dominant early-onset retinal dystrophy (Vincent et al., 2014), heterozygous p.R89G (corresponds to CRX R90) mutation in OTX2 HD causes severe ocular malformations (Ragge et al., 2005), and missense mutations of the CRX K88 and R90 homologous residues in PITX2 HD are associated with dominant Rieger syndrome (Perveen, 2000). It is likely that these mutations affect HD activity in similar ways as observed in CRX, and the exact disease manifestation is determined by cell-type- or tissue-specific mechanisms. The retina is readily accessible, and a broad range of molecular tools are available for ex vivo and in vivo manipulations. We believe that CRX is an ideal model to study the pathogenic mechanisms of HD mutations and to test therapeutic regimens, which would ultimately benefit the study of HD TFs and their associated diseases in other tissues and organs.

One limitation of this work is that effects of E80A and K88N mutations on CRX HD–DNA interactions have been evaluated at monomeric HD motifs and with homogenous protein species both in vitro and in vivo. Further evaluation of WT and mutant CRX binding at dimeric motifs will be desirable, since selected dimeric HD motifs are known to mediate HD TF interactions to ensure gene expression fidelity during development (Rister et al., 2015; Tucker and Wisdom, 1999). Relatedly, we also need to address how CRX WT and mutant E80A or K88N proteins interact at HD-binding motifs – whether they cooperate or compete with each other, whether these interactions are HD motif sequence dependent, and how gene expression is impacted by CRX cooperativity or competition. While CRX HD mediates both TF–DNA interactions and protein–protein interactions, evaluation of how E80A and K88N mutations impact CRX interaction with other important photoreceptor TFs and how perturbations in these interactions lead to disease phenotypes warrant further study.

Collectively, our findings support a unifying model in which precise CRX interaction with cis-regulatory sequences is essential for gene expression and functional maturation during photoreceptor development. Disease-associated mutations in CRX have been classified into two main groups – insertion/deletion-derived frameshift mutations in the AD and missense mutations in the HD (Tran and Chen, 2014). Prior biochemical and mouse model studies of the first group have established that AD-truncated mutant proteins abolish CRX transcriptional activity and functionally interfere with the WT allele. As a result, the mutant retinas fail to activate or maintain robust cone/rod gene expression, resulting in incomplete photoreceptor differentiation and ultimately rapid degeneration of immature photoreceptors (Furukawa et al., 1999; Tran et al., 2014). In this study, we demonstrate that missense mutations in the CRX HD, by either a loss- or gain-of-function mechanism, alter CRX target specificity and/or CRX transactivation activity. These biochemical property changes impair CRX-mediated transcriptional regulation in vivo and lead to distinct morphological and functional deficits (Figure 7A–D). Despite the difference in molecular mechanisms, both *Crx^E80A^* and *Crx^K88N^* mouse models develop whorls and rosettes in the ONL by P21, which are not observed in degenerative CRX mouse models (Tran et al., 2014), suggesting distinct pathogenic mechanisms. Future cellular biology studies are needed to understand the formation mechanisms of these unique cellular phenotypes (ONL disorganization) and their impacts on the function and survival of photoreceptors and other retinal cell types over development.

Our study here also emphasizes the importance of tailoring gene therapy regimens to tackle individual pathogenic mechanisms. For instance, while supplementing WT CRX might be sufficient to rescue a hypomorphic/loss-of-function mutant, simultaneous elimination of a gain-of-function *CRX* product would be necessary to rescue dominant mutants, as exemplified in a recent report of allele-specific gene editing to rescue dominant CRX-associated LCA7 phenotypes in a retinal organoid model (Chirco et al., 2021). We believe that this principle also applies to other dominant neurological diseases. Additionally, with the refinement of the CRX mechanistic model, when new disease mutations are identified, genetic counsellors can now provide more informed predictions of disease progression and future visual deficits. This information is important for individuals to be psychologically prepared and seek necessary assistance to improve their quality of life.

## Materials and methods

**Key resources table:** 

Reagent type (species) or resource	Designation	Source or reference	Identifiers	Additional information
Gene (*Homo sapiens*)	*CRX*	HGNC	HGNC:2383	
Gene (*M. musculus*)	*Crx*	MGI	MGI:1194883	
Strain, strain background (*Escherichia coli*)	BL21 (DE3)	MilliporeSigma	CMC0016	Electrocompetent cells
Genetic reagent (*M. musculus*)	WT (C57BL/6J)	The Jackson Laboratory	Cat #000664	
Genetic reagent (*M. musculus*)	*Crx^E80A^* (C57BL/6J)	This paper		See Materials and methods
Genetic reagent (*M. musculus*)	*Crx^K88N^* (C57BL/6J)	This paper		See Materials and methods
Genetic reagent (*M. musculus*)	*Crx^R90W^* (C57BL/6J)	Tran et al., 2014		
Cell line (*Homo sapiens*)	HEK293T	ATCC	CRL-3216	
Antibody	anti-CRX A-9 (mouse monoclonal)	Santa Cruz Biotechnology	sc-377138	ChIP: 6 µg per 400 µl reaction
Antibody	anti-CRX M02 (mouse monoclonal)	Abnova Corp.	H00001406-M02	WB: 1:1000
Antibody	anti-HDAC1 H51 (rabbit polyclonal)	Santa Cruz Biotechnology	sc-7872	WB: 1:1000
Antibody	anti-mCAR (rabbit polyclonal)	MilliporeSigma	AB15282	IH: 1:200
Antibody	anti-RHO (mouse monoclonal)	MilliporeSigma	O4886	IH: 1:200
Antibody	Anti-RXRγ Y-20 (rabbit polyclonal)	Santa Cruz Biotechnology	sc-555	IH: 1:100
Antibody	IRDye 680RD Goat anti-Rabbit IgG Secondary Antibody (mouse polyclonal)	LI-COR	926-68071	WB: 1:10,000
Antibody	IRDye 800CW Goat anti-Mouse IgG Secondary Antibody (mouse polyclonal)	LI-COR	926-32210	WB: 1:10,000
Commercial assay or kit	Amicon Ultra-0.5 Centrifugal Filter Unit	Millipore	UFC500324	
Commercial assay or kit	Dual-Luciferase Reporter Assay System	Promega	E1910	
Commercial assay or kit	GST SpinTrap	Cytiva	28952359	
Commercial assay or kit	iScript Reverse Transcription Supermix	Bio-Rad Laboratories	1708841	
Commercial assay or kit	MEGAshortscript T7 Transcription Kit	Thermo Fisher Scientific	AM1354	
Commercial assay or kit	MinElute PCR Purification Kit	QIAGEN	28006	
Commercial assay or kit	NE-PER Nuclear and Cytoplasmic Extraction Reagents	Thermo Scientific	78833	
Commercial assay or kit	Novex WedgeWell 12% Tris-Glycine Mini Protein Gels	Invitrogen	XP00122BOX	
Commercial assay or kit	NuPAGE 4 to 12%, Bis-Tris Mini Protein Gels	Invitrogen	NP0322BOX	
Commercial assay or kit	Phusion High-Fidelity PCR Master Mix with HF Buffer	New England Biolabs	M0531S	
Commercial assay or kit	SsoFast EvaGreen Supermix with Low ROX	Bio-Rad Laboratories	1725211	
Chemical compound, drug	Atropine sulfate solution	Bausch and Lomb	NDC 24208-825-55	
Chemical compound, drug	Chameleon Duo Pre-stained Protein Ladder	LI-COR	928-60000	
Chemical compound, drug	Dithiothreitol (DTT)	Bio-Rad Laboratories	1610611	
Chemical compound, drug	Exonuclease I (*E. coli*)	New England Biolabs	M0293S	
Chemical compound, drug	Gibco Dulbecco’s modified Eagle medium	Thermo Fisher Scientific	11965084	
Chemical compound, drug	Gibco fetal bovine serum	Thermo Fisher Scientific	16000044	
Chemical compound, drug	Glutathione Sepharose 4B resin	Cytiva	17075601	
Chemical compound, drug	Isopropyl-β-D-thiogalactopyranoside (IPTG)	Thermo Fisher Scientific	BP1755-10	
Chemical compound, drug	Molecular Biology Grade Water	Corning	46-000-CM	
Chemical compound, drug	Penicillin–streptomycin	Thermo Fisher Scientific	15140122	
Chemical compound, drug	Phosphate-buffered saline	Corning	46-013-CM	
Chemical compound, drug	Roche cOmplete, Mini Protease Inhibitor Cocktail	MilliporeSigma	11836153001	
Chemical compound, drug	SeeBlue Plus2 Pre-stained Protein Standard	Invitrogen	LC5925	
Chemical compound, drug	Triton X-100	Sigma-Aldrich	T9284	
Chemical compound, drug	TRIzol Reagent	Invitrogen	15596026	
Chemical compound, drug	VECTASHIELD HardSet Antifade Mounting Medium with DAPI	Vector Laboratories	H-1500-10	
Software and algorithms	bedtools (v2.27.1)	Quinlan and Hall, 2010		https://bedtools.readthedocs.io/en/latest/
Software and algorithms	Bowtie2 (v 2.3.4.1)	Langmead and Salzberg, 2012		https://bowtie-bio.sourceforge.net/bowtie2/index.shtml
Software and algorithms	BSgenome (v 1.58.0)	Pagès, 2020		https://bioconductor.org/packages/BSgenome
Software and algorithms	Clustal Omega	Goujon et al., 2010; Sievers et al., 2011		https://www.ebi.ac.uk/Tools/msa/clustalo/
Software and algorithms	DAVID (v6.8)	Sherman et al., 2022		https://david.ncifcrf.gov/
Software and algorithms	deeptools (v3.0.0)	Ramírez et al., 2016		https://deeptools.readthedocs.io/en/develop/
Software and algorithms	DEseq2 (v1.30.1)	Love et al., 2014		https://bioconductor.org/packages/DESeq2
Software and algorithms	DiffBind (v3.0.15)	Stark and Brown, 2012; Ross-Innes et al., 2012		https://bioconductor.org/packages/DiffBind
Software and algorithms	fastcluster (v1.1.26)	Müllner, 2013		http://danifold.net/fastcluster.html
Software and algorithms	FastQC (v0.11.5)	Andrews, 2010		http://www.bioinformatics.babraham.ac.uk/projects/fastqc/
Software and algorithms	GraphPad Prism 8	GraphPad Software		https://www.graphpad.com/scientific-software/prism/
Software and algorithms	GREAT (v4.0.4)	McLean et al., 2010		http://great.stanford.edu/public/html/
Software and algorithms	HOMER (v4.8)	Software and algorithms		http://homer.ucsd.edu/homer/motif/
Software and algorithms	IDR framework (v2.0.4)	Li et al., 2011		https://github.com/nboley/idr; Boley, 2017
Software and algorithms	IGV Web App	Robinson et al., 2011		https://igv.org/
Software and algorithms	Jalview (v2.11.1.7)	Waterhouse et al., 2009		https://www.jalview.org/
Software and algorithms	kallisto (v0.46.2)	Bray et al., 2016		https://github.com/pachterlab/kallisto; Pachter Lab, 2023
Software and algorithms	logomaker (v0.8)	Tareen et al., 2020		https://logomaker.readthedocs.io/en/latest/
Software and algorithms	MACS2 (v2.1.1.20160309)	Zhang et al., 2008		https://github.com/macs3-project/MACS; MACS3 project team, 2012
Software and algorithms	matplotlib (v3.5.1)	Hunter, 2007		https://matplotlib.org/
Software and algorithms	MEME Suite (v5.0.4)	Bailey et al., 2015		https://meme-suite.org/meme/index.html
Software and algorithms	rg.Mm.eg.db (v3.12.0)	Carlson, 2019		https://bioconductor.org/packages/org.Mm.eg.db
Software and algorithms	pandas (v1.4.2)	Reback et al., 2022		https://pandas.pydata.org/
Software and algorithms	Picard (v2.21.4)	Broad Institute, 2019		http://broadinstitute.github.io/picard/
Software and algorithms	python (v3.9.12)	Van Rossum and Fred, 1995		https://docs.python.org/3/reference/
Software and algorithms	R (v4.0.3)	R Core Team, 2020		
Software and algorithms	rGREAT (v1.19.2)	Gu et al., 2023		https://bioconductor.org/packages/rGREAT
Software and algorithms	samtools (v1.9)	Li et al., 2009		http://www.htslib.org/
Software and algorithms	scikit_posthocs (v0.7.0)	Terpilowski, 2019		https://scikit-posthocs.readthedocs.io/en/latest/
Software and algorithms	scipy (v1.8.1)	Virtanen et al., 2020		https://scipy.org/
Software and algorithms	seaborn (v0.11.2)	Waskom, 2021		https://seaborn.pydata.org/
Software and algorithms	Trim Galore (v0.6.1)	Felix Krueger et al., 2023		https://github.com/FelixKrueger/TrimGalore/blob/master/Docs/Trim_Galore_User_Guide.md
Software and algorithms	tximport (v1.18.0)	Soneson et al., 2015		https://bioconductor.org/packages/tximport

### Resource availability

#### Lead contact

Further information and requests for resources and reagents should be directed to and will be fulfilled by the lead contact Shiming Chen (chenshiming@wustl.edu).

#### Materials availability

All unique/stable reagents generated in this study are available from the lead contact with a completed materials transfer agreement.

#### Data and code availability

- The raw sequencing data and processed data generated in this study have been deposited at NCBI under the accession number GEO: GSE223659.
- Customized scripts and any additional information required to reproduce the analysis in this paper are available from GitHub at https://github.com/YiqiaoZHENG/CRXHD_mousemodel.

### Animal study and sample collection

#### Mutation knock-in mouse model generation

CRISPR/Cas9-based genome editing was performed to generate the *Crx^E80A^* and *Crx^K88N^* mice as previously described (Yang et al., 2014). The Cas9 guide RNAs (gRNA) were designed based on proximity to the target amino acid and was synthesized using the MEGAshortscript T7 Transcription Kit (Thermo Fisher Scientific, Waltham, MA). The gRNAs were subsequently tested for cutting efficiency in cell culture by the Washington University Genome Engineering and iPSC Center. The validated gRNA and Cas9 protein were then microinjected into the pronuclei of C57Bl/6J- 0.5-dpc (days post coitum) zygotes along with the donor DNA, a 190-bp single-stranded oligodeoxynucleotide (ssODN) carrying either the *c.239A>G* substitution for p.E80A mutation or the *c.264G>T* substitution for p.K88N mutation (Cho et al., 2009). Embryos were then transferred into the oviduct of pseudo-pregnant female. Pups were generally delivered ~20 days after microinjection. Tissues from 10-day postnatal (P10) pups were collected by toe biopsy/tail for identification of the targeted allele by restriction digest (HinfI) of PCR amplified DNA first and then confirmed by Sanger sequencing (Genewiz).

Founders carrying the correct alleles were then bred with WT C57BL/6J mice (Jackson Laboratories, Bar Harbor, ME, Strain #000664) to confirm transmission. All experimental animals used were backcrossed at least 10 generations. Genotyping of mutation knock-in mice follows cycling conditions: 95°C for 2 min, 94°C for 30 s, 60°C for 30 s, 68°C for 60 s, repeat steps 2–4 for 34 cycles, 68°C for 7 min, and hold at 4°C. After PCR reaction, the amplified DNA fragments wee digested with HinfI. Sequences of gRNAs, ssODNs, and genotyping primers can be found in Supplementary file 1c. A representative DNA gel of HinfI digested genotyping DNA fragments can be found at Figure 2—figure supplement 1B.

#### RNA-seq sample collection and library preparation

For each genotype, three biological replicates, two retinas per replicate from one male and one female mouse were analyzed. All retinas were processed for RNA simultaneously using TRIzol Reagent (Invitrogen, Waltham, MA) following the manufacturer’s protocol. The quantity and quality of the RNA were assayed using Bioanalyzer (Agilent, Santa Clara, CA). Samples with a minimum RNA integrity number score of 8.0 were then selected for library construction as previously described (Ruzycki et al., 2015).

#### Chromatin immunoprecipitation and library preparation

CRX chromatin immunoprecipitation was performed as previously published (Chen et al., 2004). Briefly, pooled nuclear extracts from six retinae were cross-linked with formaldehyde prior to immunoprecipitation with anti-CRX antibody A-9 (#sc-377138, Santa Cruz Biotechnology, Dallas, TX). Input controls were included as background. The libraries were prepared following the standard ChIP-seq protocol (Schmidt et al., 2009). The quantity and quality of the ChIP-seq libraries were assayed using Bioanalyzer (Agilent, Santa Clara, CA) prior to sequencing.

#### ERG and statistical analyses

ERGs were performed on 1-month-old mice using UTAS-E3000 Visual Electrodiagnostic System (LKC Technologies Inc, MD). Mice were dark-adapted overnight prior to the tests. Mouse body temperature was kept at 37 ± 0.5°C during the tests. Pupils were dilated with 1% atropine sulfate solution (Bausch and Lomb). Platinum 2.0 mm loop electrodes were placed on the cornea of each eye. A reference electrode was inserted under the skin of the mouse’s head and a ground electrode was placed under the skin near mouse’s tail. Retinal response to full-field light flashes (10 μs) of increasing intensity were recorded; maximum flash intensity for dark-adapted testing was 0.895 cd*s/m^2^. Following dark-adapted tests, mice were light adapted under light condition (about 29.2 cd/mm) for 10 min and exposed to 10 μs light flashes of increasing intensity; maximum flash intensity for light-adapted testing was 2.672 cd*s/m^2^. ERG responses of biological replicates were recorded, averaged, and analyzed using GraphPad Prism 8 (GraphPad Software, CA). The mean peak amplitudes of dark-adapted A- and B-waves and light-adapted B-waves were plotted against log values of light intensities (cd*s/m^2^). The statistics were obtained by two-way analysis of variance (ANOVA) with multiple pairwise comparisons (Tukey’s).

#### Histology and immunohistology chemistry

Enucleated eyes were fixed at 4°C overnight for paraffin-embedded sections. Each retinal cross-section was cut 5 µm thick on a microtome. H&E staining was performed to examine retinal morphology. For IHC staining, sections firstly went through antigen retrieval with citrate buffer, and blocked with a blocking buffer of 5% donkey serum, 1% bovine serum albumin, 0.1% Triton X-100 in 1× phosphate-buffered saline (PBS) (pH 7.4) for 1 hr. Sections were then incubated with primary antibodies at 4°C overnight. Sections were washed with 1× PBS containing 0.01% Triton X-100 (PBST) for 30 min, and then incubated with specific secondary antibodies for 1 hr. Primary and secondary antibodies were applied with optimal dilution ratios. All slides were mounted with VECTASHIELD HardSet Antifade Mounting Medium with DAPI (Vector Laboratories, Inc, CA). All images were taken on a Leica DB5500 microscope. All images were acquired at 1000 µm from ONH for ≥P21 samples and at 500 µm from ONH for P0, P3, and P10 samples.

### Biochemistry

#### Protein expression

Expression plasmids for GST-WT, E80A, and R90W HDs were published previously (Chen et al., 2002). Plasmid for GST- K88N HD was generated by site-directed mutagenesis from the pGEX4T2-CRX WT HD backbone. In vivo protein expression and purification were done as previously described (Chen et al., 2002). Briefly, 0.05 mM isopropyl-β-D-thiogalactopyranoside was added to *E. coli* BL-21 (DE3) cell cultures containing different CRX HD constructs at OD_600_ = 0.6. The cultures were incubated for 2 hr or until OD_600_ = 2.0 at 34°C and the cells were collected by centrifugation at 6000 rpm and 4°C for 15 min. Cell pellets were resuspended in 1× PBS (Corning, Corning, NY) and then lysed by sonication. 5 mM dithiothreitol (DTT) (Bio-Rad Laboratories, Inc, Hercules, CA) and 1% Triton X-100 (MilliporeSigma, Burlington, MA) was then added, and the mixtures were incubated with gentle shaking at 4°C for 30 min to maximize protein extraction. The separation of proteins from the cellular debris were then performed by centrifugation at 15,000 rpm for 10 min and filtered through a 0.45-μm membrane. Glutathione Sepharose 4B resin (Cytiva, Marlborough, MA) was first equilibrated with PBS before adding to the supernatant. 5× Halt Protease Inhibitor Cocktail and phenylmethylsulfonyl fluoride (PMSF) was added to minimize degradation. The mixtures were incubated with gentle shaking at 4°C overnight before loading on GST Spintrap columns (Cytiva, Marlborough, MA). The peptides were eluted following the manufacturer’s protocol and buffer exchanged into CRX-binding buffer (Lee et al., 2010) using Amicon centrifugal filters (MilliporeSigma, Burlington, MA). The protein stock was supplemented with 10% glycerol before aliquoted and stored at −80°C.

#### Protein quantification and visualization

The size and integrity of purified GST-CRX HDs were visualized with a native 12% Tris-Glycine sodium dodecyl sulfate–polyacrylamide gel electrophoresis (SDS–PAGE) gel in the absence any reducing agent. Protein concentration was measured by NanoDrop Oneᶜ Microvolume UV-Vis Spectrophotometers (Thermo Fisher Scientific, Waltham, MA) and calculated using the equation: *C* = (1.55 * *A*_280_) − (0.76 * *A*_260_), where *C* is the concentration of the protein in mg/ml, *A*_280_ and *A*_260_ are the absorbance of protein samples at 280 and 260 nm, respectively (Roy et al., 2017). The protein concentrations obtained with this method were comparable with Bicinchoninic Acid (BCA) protein quantification assays.

#### Spec-seq library synthesis and purification

Single-stranded Spec-seq library templates and IRDye 700-labeled reverse complement primers (Supplementary file 1b) were ordered directly from Integrated DNA Technologies (IDT, Coralville, Iowa). The synthesis and purification of the double-stranded libraries followed previously published protocols (Zuo et al., 2017; Zuo and Stormo, 2014; Roy et al., 2017). Briefly, 100 pmol of template oligos and 125 pmol IRDye 700-labeled reverse complement primer F1 were mixed in Phusion High-Fidelity PCR Master Mix (NEB, Ipswich, MA). A 15-s denaturing at 95°C following a 10-min extension at 52°C afforded duplex DNAs. Subsequently, the mixture was treated with 1 µl Exonuclease I (NEB, Ipswich, MA) to remove excess ssDNA. The libraries were purified by MinElute PCR Purification Kit (QIAGEN, Hilden, Germany) and eluted in molecular biology graded water (Corning, Corning, NY).

#### EMSA and sample preparation for sequencing

The protein–DNA-binding reactions was done in 1× CRX-binding buffer (60 mM KCl, 25 mM (4-(2-hydroxyethyl)-1-piperazineethanesulfonic acid) HEPES, 5% glycerol, 1 mM DTT) (Lee et al., 2010). A fixed amount (Supplementary file 1b) of IRDye-labeled DNA libraries were incubated on ice for 30 min with varying concentrations of WT or mutant peptides in 20 μl reaction volume. The reaction mixtures were run at 4°C in native 12% Tris-Glycine PAGE gel (Invitrogen, Waltham, MA) at 160 V for 40 min. The IRDye-labeled DNA fragments in the bound and unbound fractions were visualized by Odyssey CLx and Fc Imaging Systems (LI-COR, Inc, Lincoln, NE). The visible bands were excised from the gels and DNAs were extracted with acrylamide extraction buffer (100 mM NH_4_OAc, 10 mM Mg(OAc)_2_, 0.1% SDS) then purified with MinElute PCR Purification Kit (QIAGEN, Hilden, Germany). The DNAs were amplified, barcoded by indexed Illumina primers. All indexed libraries were then pooled and sequenced on a single 1 × 50 bp Miseq run at DNA Sequencing Innovation Lab at the Center for Genome Sciences & Systems Biology (CGS&SB, WashU).

#### qRT-PCR

For each replicate, RNA from two retinae of a mouse was extracted using the NucleoSpin RNA kits (Takara Bio USA, Inc, San Jose, CA). RNA sample concentration and quality were determined with NanoDrop Oneᶜ Microvolume UV-Vis Spectrophotometers (Thermo Fisher Scientific, Waltham, MA). 1 μg of RNA was used for cDNA synthesis with iScript cDNA Synthesis Kits (Bio-Rad, Hercules, CA) in a 20-μl reaction volume. Primers used in this study are listed in Supplementary file 1b. qRT-PCR reactions were assembled using SsoFast EvaGreen Supermix with Low ROX (Bio-Rad Laboratories, Inc, Hercules, CA) following the manufacturer’s protocol. Data were obtained from Bio-Rad CFX96 Thermal Cycler following a three-step protocol: 1 cycle of 95°C 3 min, 40 cycles of 95°C 10 s, and 60°C 30 s. Data were exported and further processed with customized python script.

#### Western blot

Experiments were performed using two biological replicates with two retinas for each replicate. Nuclear extracts were prepared using the NE-PER Nuclear and Cytoplasmic Extraction Reagents (Thermo Fisher Scientific, Waltham, MA) following manufacturer’s instructions. 1× Roche cOmplete Mini Protease Inhibitor Cocktail (MilliporeSigma, Burlington, MA) was supplemented in all extraction reagents. 5 mM of DTT was added immediately before sample denaturing and protein was separated by running on Invitrogen NuPAGE Novex 4–12% Bis-Tris MiniGels (Invitrogen, Waltham, MA). Membrane transfer was done with the Blot mini blot module (Invitrogen, Waltham, MA) following the manufacturer’s protocol. Membrane was probed with mouse monoclonal anti-CRX antibody M02 (1:1000, Abnova Corp, Taipei City, Taiwan) and rabbit polyclonal anti-HDAC1 antibody H51 (1:1000, Santa Cruz Biotechnology, Dallas, TX), visualized with IRDye 680RD goat anti-rabbit IgG and IRDye 800CW goat anti-mouse IgG secondary antibodies (1:10,000, LI-COR, Inc, Lincoln, NE). The membrane was then imaged using Odyssey CLx and Fc Imaging Systems (LI-COR, Inc, Lincoln, NE).

#### Cell line transient transfection luciferase reporter assays

HEK293T cells (CRL-3216) were obtained directly from ATCC (American Type Culture Collection). The cells were used within 1 year of purchase and tested negative for mycoplasma contamination. Cells are cultured in Dulbecco’s modified Eagle medium supplemented with 10% fetal bovine serum and penicillin–streptomycin following the manufacturer’s protocol. Cells were transfected with calcium phosphate transfection protocol in 6-well plates as previously described (Chen et al., 2002; Tran et al., 2014). Experimental plasmids and usage amount are described in Supplementary file 1b. Typically, 48 hr after transfection, cells were harvested, digested, and assayed for luciferase activity using Dual-Luciferase Reporter Assay System (Promega, Madison, WI) following the manufacturer’s protocol. Data were collected using TD-20/20 Luminometer (Turner Designs, East Lyme, CT) and further processed with customized python scripts.

### Data analysis

#### HD sequence alignment

The full-length protein sequences for the selected TFs were first aligned with Clustal Omega (EMBL-EBI, UK). Aligned sequences of the third HD helix were then extracted to generate Figure 1B using Jalview (v2.11.1.7). A list of the accession numbers for the selected TFs can be found in Supplementary file 1a.

#### Determination of relative binding affinity with Spec-seq

For a biomolecular interaction between a protein *P* and a particular DNA sequence, *S_i_*, the interaction can be diagrammed as:

(1) $$P+S_{i}\;⇌P\cdot S_{i}$$

where $P{∙S}_{i}$ refers to the protein–DNA complex. The affinity of the protein *P* to sequence *S_i_* is defined as the association constant *K_A_*, or its reciprocal, the dissociation constant *K_D_*. The *K_A_* of the protein–DNA interaction is determined by measuring the equilibrium concentrations of each reactant and the complex:

(2) $$K_{A}\left(S_{i}\right)=\;\frac{\left[P\cdot S_{i}\right]\;}{\left[P\right]\cdot \left[S_{i}\right]}$$

where […] refers to concentrations. As in a typical Spec-seq experiment, thousands of different DNA sequences compete for the same pool of proteins, their relative binding affinities (the ratio of their *K_A_*) can be determined by measuring the concentrations of each sequence in the bound and unbound fractions without measuring the free protein concentrations, which is often the most difficult to measure accurately:

(3) $$K_{A}\left(S_{1}\right)\;:K_{A}\left(S_{2}\right)\;:\ldots \;:K_{A}\left(S_{n}\right)=\;\frac{\left[P\cdot S_{1}\right]\;}{\left[S_{1}\right]}\;:\;\frac{\left[P\cdot S_{2}\right]\;}{\left[S_{2}\right]}\;:\ldots \;:\;\frac{\left[P\cdot S_{n}\right]\;}{\left[S_{n}\right]}$$

In a binding reaction involving TF and a library of DNAs, the concentration of bound and unbound species are directly proportional to the number of individual DNA molecules in each fraction which can be obtained directly from sequencing data. With enough counts in each fraction, we can accurately estimate the ratios of concentrations from counts with the relationship:

(4) $$\frac{\left[P\cdot S_{i}\right]\;}{\left[P\cdot S_{j}\right]}\approx \;\frac{N_{B}\left(S_{i}\right)}{N_{B}\left(S_{j}\right)}\;\;and\;\frac{\left[S_{i}\right]\;}{\left[S_{j}\right]}\approx \;\frac{N_{U}\left(S_{i}\right)}{N_{U}\left(S_{j}\right)}$$

where *N_U_* denotes counts in the unbound fraction and *N_B_* denotes counts in the bound fraction. Therefore, the binding affinity of a sequence variant *S_x_* relative to the reference sequence *S_ref_* can be calculated by:

(5) $$\frac{K_{A}\left(S_{x}\right)}{K_{A}\left(S_{ref}\right)}\;\approx \;\frac{\left[P\cdot S_{x}\right]\;}{\left[P\cdot S_{ref}\right]}\frac{\left[S_{ref}\right]\;}{\left[S_{x}\right]}\;\approx \;\frac{N_{B}\left(S_{x}\right)}{N_{B}\left(S_{ref}\right)}\frac{N_{U}\left(S_{ref}\right)}{N_{U}\left(S_{x}\right)}$$

The natural logarithms of these ratios are the relative binding free energies in the units of kcal/mol. The relative free energy of the reference site for each CRX HD was set to 0.

#### Spec-seq data analysis and energy logo visualization

The sequencing results were first filtered and sorted based on conserved regions and barcodes. Reads with any mismatch in the conserved regions were discarded prior to further analysis as described previously (Stormo et al., 2015; Zuo and Stormo, 2014; Roy et al., 2017). The ratio of individual sequence in bound and unbound reads was calculated as a measurement of relative binding affinity (Equation 5Equation 5) compared to the consensus sequence. The relative binding energy was then derived from the natural logarithm of the relative binding affinity and represented in kcal/mol units.

For WT CRX HD and all the mutants, the energy weight matrices (ePWMs) were generated based on the regression of the TF’s binding energy to its reference sequence. Only sequences within two mismatches to the reference were used to generate the ePWMs. Energy logos were generated from ePWMs after normalizing the sum of energy on each position to 0 and the negative energy values were plotted such that preferred bases are on top. The sequence logos were generated from ePWMs with python package logomaker (v0.8). The ePWMs for all CRX HDs are listed in Supplementary file 1c.

#### ChIP-seq data analysis

2 × 150 bp reads from Illumina NovaSeq were obtained for all samples with a minimum depth of 22 M reads at Novogene (Beijing, China). For each sample, reads from two sequencing lanes were first concatenated and run through Trim Galore (v0.6.1) (Felix Krueger et al., 2023) to remove adapter sequences and then QC by FastQC (v0.11.5) (Andrews, 2010). The trimmed reads were then mapped to the mm10 genome using Bowtie2 (v 2.3.4.1) (Langmead and Salzberg, 2012) with parameters -X 2000 --very-sensitive. Only uniquely mapped and properly paired reads were retained with samtools (v1.9) (Li et al., 2009) with parameters -f 0x2 -q 30. Mitochondria reads were removed with samtools (v1.9) (Li et al., 2009). Duplicated reads were marked and removed with Picard (v2.21.4) (Broad Institute, 2019). Last, reads mapped to the mm10 blacklist regions were removed by bedtools (v2.27.1) (Quinlan and Hall, 2010) by intersect -v. bigWig files were generated with deeptools (v3.0.0) (Ramírez et al., 2016) with command bamCoverage --binSize 10 -e --normalizeUsing CPM and visualized on IGV Web App (Robinson et al., 2011). For each genotype, an average binding intensity bigWig file from two replicates was generated with deeptools (v3.0.0) (Ramírez et al., 2016) command bamCompare –operation mean with default parameters.

Peak-calling was done with MACS2 (v2.1.1.20160309) (Zhang et al., 2008) on individual replicate with the default parameters. For each genotype, we then generated a genotype-specific high confidence peakset by intersection of peaks called in two replicates. IDR framework (v2.0.4) (Li et al., 2011) were used to generate quality metrics for the processed ChIPseq data. R package DiffBind (v3.0.15) (Stark and Brown, 2012; Ross-Innes et al., 2012) and DEseq2 (v1.30.1) (Love et al., 2014) were then used to re-center peaks to ±200 bp regions surrounding summit, generate normalized binding intensity matrix, and differential binding matrix. We defined differentially bound peaks between each mutant and WT sample if the absolute log_2_FC is more than 1.0, corresponding to twofold, and the false discovery rate (FDR) is smaller than 5e−2.

To associate peaks to genes, we used Genomic Regions Enrichment of Annotations Tool (GREAT v4.0.4) (McLean et al., 2010) through the R package rGREAT (v1.19.2) (Gu et al., 2023). Each peak was assigned to the closest TSS within 100 kb.

#### Binding intensity heatmap and clustering

To generate the binding intensity heatmap in Figure 2A, we first compiled the genotype-specific high confidence peakset for all genotypes into a single consensus peakset and only peaks with at least 5 cpm in all genotypes were retained. Python package fastcluster (v1.1.26) (Müllner, 2013) was used to perform hierarchical clustering of the consensus peakset intensity matrix with parameters method=’single', metric='euclidean'. The genomic regions corresponding to the two major clusters were exported and used to generate binding intensity heatmaps with deeptools (v3.0.0) (Ramírez et al., 2016).

#### Genomic region enrichment of CRX peaks

Peak annotation in Figure 2D were obtained using annotatePeaks.pl from HOMER (v4.8).

#### De novo motif searching

The mm10 fasta sequences for each genotype-specific peaks were obtained using R package BSgenome (v 1.58.0) (Pagès, 2020). De novo motif enrichment analysis for each set of sequences was then performed with MEME-ChIP in MEME Suite (v5.0.4) (Bailey et al., 2015) using order 1 Markov background model and default parameters. Since HD motifs are relatively short and can be repetitive (e.g., K88N motif), we reported DREME (Bailey, 2011) found motifs for Figure 2, which is more sensitive than MEME to find short, repetitive motifs.

#### RNA-seq data analysis

2 × 150 bp reads from Illumina NovaSeq were obtained for all samples with a minimum depth of 17 M reads at Novogene (Beijing, China). Sequencing reads were first run through Trim Galore (v0.6.1) (Felix Krueger et al., 2023) to remove adapter sequences and then QC by FastQC (v0.11.5) (Andrews, 2010). Trimmed reads were then mapped to the mm10 genome and quantified with kallisto (v0.46.2) (Bray et al., 2016). Kallisto output transcript-level abundance matrices were then imported and summarized into gene-level matrices with R package tximport (v1.18.0) (Soneson et al., 2015). DEseq2 (v1.30.1) (Love et al., 2014) was then used for normalization and differential expression analysis. The normalized count and differential expression matrices were then exported and further processed with customized python scripts.

We defined differentially expressed genes between each mutant and WT sample if the absolute log_2_FC is more than 1.0, corresponding to twofold, and the FDR is smaller than 1e−2. For comparison between heterozygous and homozygous mutants, we first filtered genes with at least 5 cpm and then those that were called differentially expressed compared with WT in at least one mutant genotype. We retrieved gene names in Supplementary file 1e–g from the Database for Annotation, Visualization and Integrated Discovery (DAVID, v6.8) (Sherman et al., 2022).

#### Definition of CRX-dependent and -independent gene set

We first identified CRX peaks that were bound in the WT sample but lost in *R90W/W* sample (log_2_FC <−1 and FDR <5e−2). This yielded a total of 7677 peaks. We then found the genes associated with these peaks. We defined a gene to be CRX-dependent activated if its expression was down in adult (P21) *R90W/W* RNA-seq sample (log_2_FC <−0.6 and FDR <1e−5). Similarly, a gene is defined as CRX-dependent suppressed if its expression was up in adult *R90W/W* RNA-seq sample (log_2_FC >0.6 and FDR <1e−5). A gene is defined as CRX independent if its expression was not significantly affected in adult *R90W/W* RNA-seq samples. There were 617 CRX-dependent activated, 135 CRX-dependent suppressed, and 5565 CRX-independent genes. Manual inspection of the CRX-dependent suppressed genes revealed no clear association with photoreceptor development. Therefore, we did not further pursue this gene set. The complete list of CRX-dependent activated genes that showed differential expression in at least one of the HD mutant retinas can be found in Supplementary file 1e. The lists for CRX-independent genes that showed differential expression in *Crx^E80A^* or *Crx^K88N^* mutant retinas can be found in Supplementary file 1f, g, respectively.

#### GO analysis

GO analysis in Figure 3—figure supplement 1B, E and Figure 3—figure supplement 2F was performed using R package clusterProfiler (v4.0.5) (Yu et al., 2012; Wu et al., 2021) with the genome-wide annotation package org.Mm.eg.db (v3.12.0) (Carlson, 2019). Redundant enriched GO terms were removed using simplify() function with parameters cutoff = 0.7, by="p.adjust". The enrichment analysis results were then exported in table format and further processed for plotting with python.

#### Aldiri et al. RNA-seq data re-analysis

The RNA-seq data from Aldiri et al., 2017 were obtained from GEO under accession numbers GSE87064. The reads were processed similarly as all other RNA-seq data generated in this study. For Figure 3—figure supplement 1C, F, Figure 3—figure supplement 2C, E, Figure 4—figure supplement 1A, B, expression row *z*-scores were calculated using average cpm from replicates at each age.

#### Statistical analysis

One-way ANOVA with Turkey honestly significant difference test in Figure 6A, Figure 2—figure supplement 1C, and Figure 6—figure supplement 1D was performed with python packages scipy (v1.8.1) (Virtanen et al., 2020) and scikit_posthocs (v0.7.0) (Terpilowski, 2019). Two-sided Mann–Whitney *U*-test in Figure 3—figure supplement 2A was performed with python package scipy (v1.8.1) (Virtanen et al., 2020).

## Data Availability

The raw sequencing data and processed data generated in this study have been deposited at NCBI under the accession number GEO: GSE223659. Customized scripts and any additional information required to reproduce the analysis in this paper are available from GitHub at https://github.com/YiqiaoZHENG/CRXHD_mousemodel copy archived at Zheng, 2023. The following dataset was generated: ZhengY SunC ZhangX RuzyckiP ChenS 2023Missense mutations in CRX homeodomain cause dominant retinopathies through two distinct mechanismsNCBI Gene Expression OmnibusGSE22365910.7554/eLife.8714737963072 The following previously published dataset was used: AldiriI XuB WangL ChenX 2017The Dynamic Epigenetic Landscape of the Retina During Development, Reprogramming, and TumorigenesisNCBI Gene Expression OmnibusGSE8706410.1016/j.neuron.2017.04.022PMC550851728472656

## Additional files

Supplementary file 1. Supplementary materials. (a) Homeodomain transcription factor (HD TF) accession numbers (related to Figure 1B). (b) Plasmids and primers for biochemistry experiments. (c) Spec-seq ePWMs (related to Figure 1G, K–M, and Figure 1—figure supplement 2I–L). (d) DNA sequences for mutation knock-in mice generation. (e) CRX-dependent activated genes differentially expressed in at least one mutant mouse model (ordered as in Figure 3B). (f) CRX-independent genes mis-regulated in *Crx^E80A^* mutants (ordered as in Figure 3—figure supplement 2C). (g) CRX-independent genes mis-regulated in *Crx^K88N^* mutants (ordered as in Figure 3—figure supplement 2E). (h) Annotation for phototransduction genes in Figure 4B. (i) Quantification and statistical analysis (related Figure 2—figure supplement 1C and Figure 3—figure supplement 2A)

MDAR checklist
