# Supplementary material for: Missense mutations in CRX homeodomain cause dominant retinopathies through two distinct mechanisms
Source: eLife. 2023 Nov 14;12:RP87147. doi: 10.7554/eLife.87147 (PMC10645426; doi:10.7554/eLife.87147)
Supplement: Figure 1—source data 1. [file elife-87147-fig1-data1.pdf]

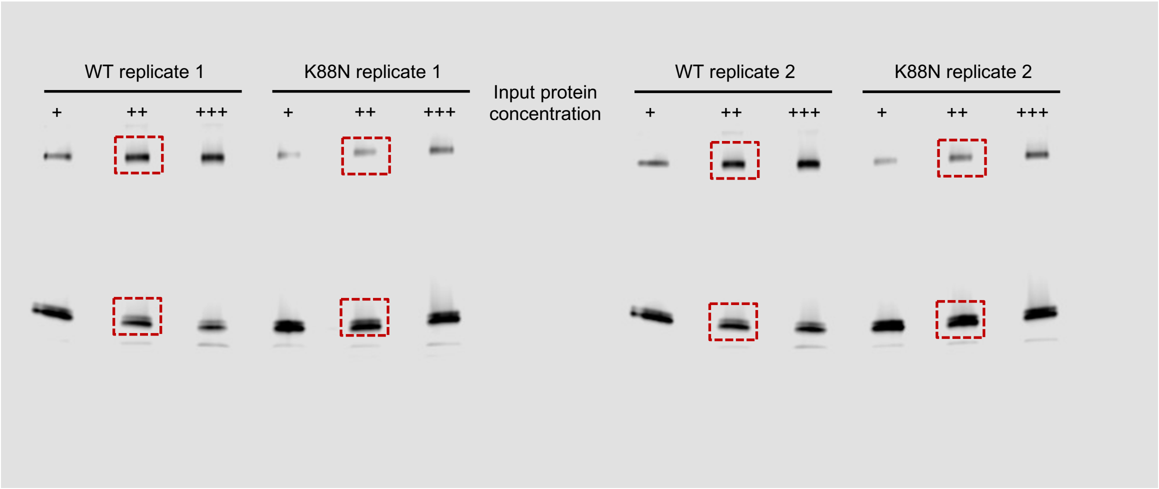

Figure 1 – source data 1. Unprocessed Spec-seq EMSA gel image for WT and K88N HD. DNA in bands highlighted by dashed rectangles were eluted and used for generating data in this study.
