# Supplementary material for: Missense mutations in CRX homeodomain cause dominant retinopathies through two distinct mechanisms
Source: eLife. 2023 Nov 14;12:RP87147. doi: 10.7554/eLife.87147 (PMC10645426; doi:10.7554/eLife.87147)
Supplement: Figure 1—figure supplement 2—source data 1. [file elife-87147-fig1-figsupp2-data1.pdf]

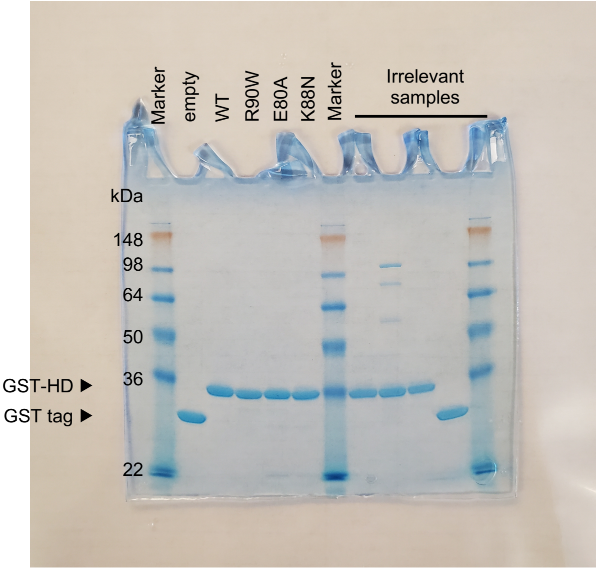

Figure 1 – figure supplement 2 - source data. Unprocessed SDS-PAGE gel image of column-purified GST-HDs. M: SeeBlue™ Plus2 Pre-stained Protein Standard (Invitrogen, LC5925).
