# Supplementary material for: Missense mutations in CRX homeodomain cause dominant retinopathies through two distinct mechanisms
Source: eLife. 2023 Nov 14;12:RP87147. doi: 10.7554/eLife.87147 (PMC10645426; doi:10.7554/eLife.87147)
Supplement: Figure 2—figure supplement 1—source data 1. [file elife-87147-fig2-figsupp1-data1.pdf]

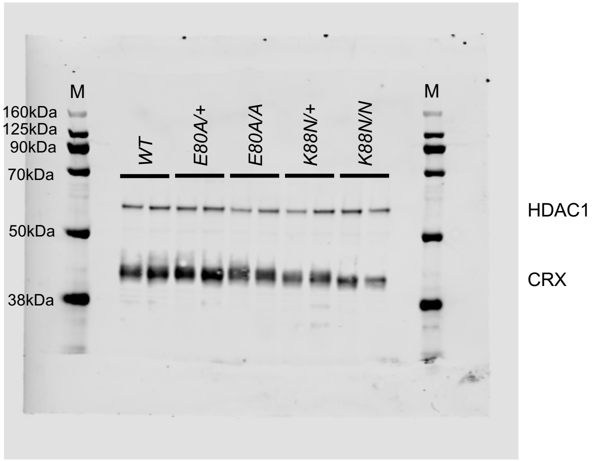

Figure 2 – figure supplement 1 – source data. Unprocessed immunoblot of P14 retina nuclear extracts stained with anti-CRX and anti-HDAC1 antibodies. M: Chameleon® Duo Pre-stained Protein Ladder (LI-COR, 928-60000).
